# Genotype, Ethnicity, and Drug–Drug Interaction Modeling as Means of Verifying Transporter Biomarker PBPK Model: The Coproporphyrin‐I Story

**DOI:** 10.1002/psp4.70008

**Published:** 2025-03-10

**Authors:** Yuki Ujihira, Shawn Pei Feng Tan, Daniel Scotcher, Aleksandra Galetin

**Affiliations:** ^1^ Centre for Applied Pharmacokinetic Research, School of Health Sciences University of Manchester Manchester UK; ^2^ Laboratory for Safety Assessment and ADME, Pharmaceuticals Research Center Asahi Kasei Pharma Corporation Shizuoka Japan

**Keywords:** biomarker, drug–drug interaction, PBPK, transporter

## Abstract

Coproporphyrin‐I (CP‐I) is a selective endogenous biomarker of organic anion‐transporting polypeptide (OATP)1B. Multiple CP‐I PBPK models with differing input parameters have been reported so far. This study proposed a harmonized CP‐I PBPK model and evaluated its ability to predict the effect of ethnicity, *SLCO1B1* genotype c.521T>C, and sex on CP‐I baseline and CP‐I‐drug interactions using the largest clinical dataset to date. The CP‐I PBPK model successfully predicted CP‐I plasma baseline from 731 subjects, with 97% of predictions within 1.5‐fold of the observed data. Prediction of weak, moderate, and strong OATP1B‐mediated interactions with probenecid, low‐dose cyclosporine, and rifampicin, respectively, was evaluated with 21 datasets. Overall, > 76% of CP‐I *C*_max_ *R* and AUCR were predicted within the Guest criterion. In vivo OATP1B *K*_*i*_ estimated by the biomarker model was up to ninefold lower compared to in vitro values. Sensitivity analyses showed differences in estimated in vivo *K*_*i*_ depending on the assumed contribution of non‐inhibited/parallel pathway (renal) for CP‐I (0%–15%), highlighting the need to consider this factor when using biomarker PBPK models for such purposes. Finally, the appropriate metric for monitoring CP‐I was evaluated for inhibitors with different potency and PK relative to CP‐I. In the case of strong/moderate OATP1B inhibitors with short *t*_1/2_, *C*_max_ *R* was the most sensitive metric for monitoring CP‐I OATP1B interactions, whereas both *C*_max_ *R* and AUCR were applicable for inhibitors with long *t*_1/2_. The current study provides a harmonized CP‐I PBPK model, together with recommendations to support the optimal design of prospective clinical trials for the assessment of OATP1B‐mediated DDIs using this biomarker.

**Summary**

- What is the current knowledge on the topic?
  ○ Coproporphyrin‐I (CP‐I) is a sensitive endogenous biomarker of organic anion‐transporting polypeptide (OATP)1B for early clinical monitoring of OATP1B‐mediated DDIs.
- What question did this study address?
  ○ Development of a harmonized CP‐I PBPK model. Critical evaluation of its ability to predict the effects of ethnicity, *SLCO1B1* c.521T>C, and sex on CP‐I baseline and CP‐I–drug interactions using the largest clinical dataset to date.
- What does this study add to our knowledge?
  ○ Credibility analysis of the biomarker model. Sensitivity analyses highlighted the importance of the assumed contribution of hepatic versus renal elimination (differences in fT_OATP1B1_) in the CP‐I model on the estimated in vivo *K*_*i*_ when the CP‐I model is used for such purposes. The appropriate metric (*C*_max_ *R* or AUCR) for monitoring CP‐I depends on the potency of the OATP1B inhibitor and its PK relative to CP‐I.
- How might this change clinical pharmacology or translational science?
  ○ The study provides a harmonized CP‐I PBPK model, together with recommendations to support the design of prospective clinical trials. The use of CP‐I *C*_max_ *R* as a more sensitive metric is recommended for the risk assessment of OATP1B‐mediated DDIs.

## 1 Introduction

Organic anion‐transporting polypeptide (OATP)1B1/3 transporters facilitate hepatic uptake of many anionic drugs and endogenous compounds (e.g., coproporphyrin I, CP‐I). Various drugs, including rifampicin and cyclosporine, inhibit these transporters, resulting in increased plasma concentrations of OATP1B substrates when administered together [1]. *SLCO1B1* c.521T>C genetic polymorphism results in differences in adverse drug effects and magnitude of OATP1B1‐mediated drug–drug interactions (DDIs) [2, 3, 4, 5]. In addition, ethnic differences and lower OATP1B1 expression have been reported in the Japanese population [6, 7, 8].

Initial assessment of transporter‐mediated DDIs in accordance with regulatory guidelines relies on in vitro inhibition constant (*K*_*i*_), together with inhibitor drug concentrations obtained in early clinical phases [9, 10, 11]. This basic approach aims to minimize false‐negative predictions but often results in high false‐positive rates, potentially leading to unnecessary follow‐up clinical DDI studies [12, 13]. In recent years, alternative approaches, such as monitoring endogenous biomarkers of relevant transporters in the early clinical stages, have gained attention [14, 15, 16]. Examples include CP‐I (OATP1B1/3 biomarker) and 4‐pyridoxic acid (biomarker for organic anion transporter (OAT)1/3) [17, 18, 19, 20, 21].

**FIGURE 1:**
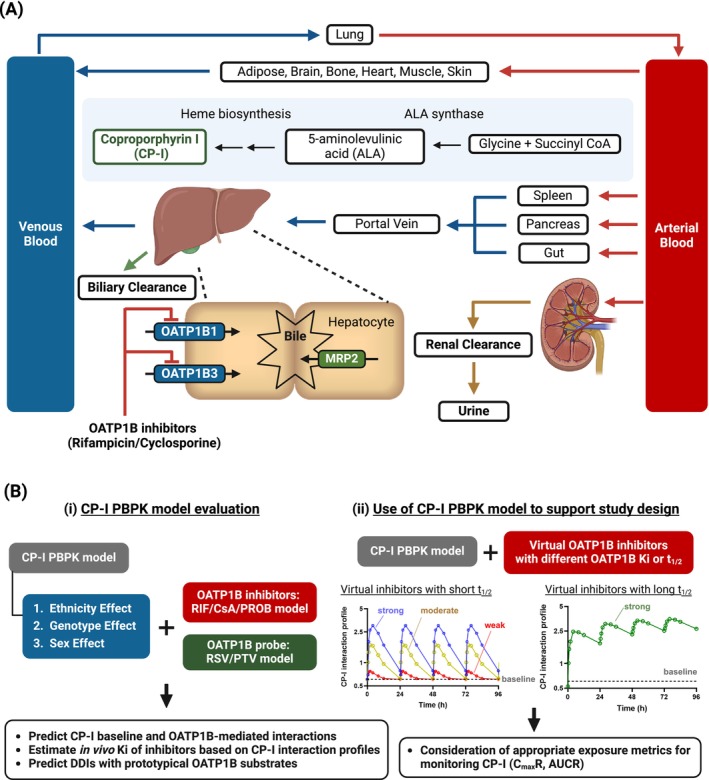
Schematic representation of the CP‐I PBPK model and an overview of this study. (A) Physiologically‐based pharmacokinetic model incorporating the biosynthesis and elimination of Coproporphyrin (CP‐I). CP‐I is a byproduct of heme synthesis, and the existing models assume that CP‐I is synthesized entirely in the bloodstream. CP‐I is eliminated via biliary and renal excretion. In the liver, the organic anion transporting polypeptides (OATP) 1B1/3 actively transport CP‐I from the portal blood into hepatocytes, after which CP‐I is secreted into the bile by multidrug resistance‐associated protein 2 (MRP2). (B) Overall evaluation flow of the CP‐I PBPK models in this study. (i) Evaluation of the CP‐I PBPK model and its ability to predict the effects of different ethnicities, *SLCO1B1* c.521T>C genotype, and sex on CP‐I baseline and OATP1B‐mediated CP‐I interactions. A summary of clinical CP‐I data is provided in Tables S1 and S3. The predictive accuracy of DDIs of prototypical OATP1B probes and a dose range of rifampicin, cyclosporine, and probenecid was investigated using in vitro and in vivo *K*_*i*_ of inhibitors (estimated from CP‐I interaction profiles). (ii) Evaluation of study design for monitoring CP‐I in phase I study. Virtual OATP1B inhibitors with different *K*_*i*_ (weak to strong) and *t*_1/2_ (short to long) were created, and the selection of appropriate exposure metrics (*C*_max_ *R* or AUCR) for monitoring CP‐I was evaluated using the CP‐I PBPK model. RIF, CsA, and PROB represent rifampicin, cyclosporine, and probenecid, respectively. RSV and PTV represent rosuvastatin and pitavastatin, respectively. These figures were created using BioRender.com.

CP‐I is a metabolically stable byproduct of heme syntheses, eliminated via biliary and renal excretion [17, 22]. CP‐I is a sensitive and selective endogenous biomarker of OATP1B transporters (Figure 1A) and its plasma concentrations have been reported to increase after the administration of OATP1B inhibitors [3, 22, 23, 24, 25]. CP‐I exhibits stable plasma levels with minimal diurnal variation, but interindividual variability in CP‐I baseline across different ethnicities, *SLCO1B1* c.521T>C genotype, and sex has been reported [3, 19, 23, 26, 27, 28].

Given high selectivity, sensitivity, multiple modeling efforts, and applications of CP‐I as a predictive OATP1B1 biomarker, it is classified as a Tier 1 biomarker by the International Transporter Consortium (ITC) [14], and monitoring CP‐I plasma concentrations in early phase I single or multiple dose escalation studies is recommended [9, 14, 15, 16]. Both the ITC and IQ Consortium have provided decision trees recommending a cut‐off value of 1.25‐fold change in CP‐I C_max_ as a trigger for further investigation of OATP1B DDI, either via modeling or by subsequent dedicated clinical studies with the OATP1B probe [9, 14, 15, 16].

Physiologically‐ based pharmacokinetic (PBPK) modeling is widely recognized as a powerful tool for PK and DDI predictions, and it allows consideration of the effects of genotype, ethnicity, and other covariates on drug exposure [19, 29, 30, 31, 32]. In recent years, the advantages of CP‐I measurement for DDI risk assessment have been demonstrated through different modeling approaches ranging from population PK (pop PK) to PBPK modeling [17, 18, 19, 25, 33, 34, 35, 36]. Initial CP‐I models explored the contribution of hepatic and renal elimination and investigated model sensitivity to the synthesis site [17, 19]. Although multiple CP‐I models have been reported, these models have primarily focused on the prediction of specific scenarios/populations in isolation but have not considered the effect of ethnicity, genotype, and sex (in isolation and combined) on CP‐I baseline and the magnitude of OATP1B‐mediated DDIs. In addition, modeling of CP‐I interaction data has been increasingly used to estimate in vivo OATP1B1 *K*_*i*_, with the aim of improving the accuracy of subsequent DDI predictions with OATP1B substrates. This approach was illustrated initially for rifampicin using population PK modeling [17], and subsequently for additional OATP1B1 inhibitors using PBPK modeling [18].

In this study, we aimed to provide a harmonized CP‐I PBPK model. To evaluate its robustness, we collected the largest CP‐I clinical data reported in individuals with different ethnicities, *SLCO1B1* c.521T>C genotype, and sex, both at the baseline level and in the case of OATP1B‐mediated interactions (Figure 1B). Our previously developed reduced CP‐I PBPK model [19] was modified to capture the recently reported contribution of OATP1B1 and OATP1B3 to hepatic uptake of CP‐I [37] and to address inconsistencies in the contribution of renal elimination across published CP‐I PBPK models. The predictive accuracy of the model was evaluated against its ability to predict the impact of these three covariates on CP‐I baseline and CP‐I interactions with a wide range of doses of rifampicin, cyclosporine, and probenecid. Sensitivity analyses were performed on the parameters contributing to differences in estimated in vivo OATP1B *K*_*i*_ using the CP‐I PBPK model. Finally, modeling and simulation were used to investigate the appropriate metrics for monitoring CP‐I in the presence of inhibitors of different potency and *t*_1/2_, with the aim of supporting the design of prospective clinical trials with OATP1B inhibitors.

## 2 Methods

### 2.1 Collation of Published CP‐I Clinical Data

Recent meta‐analysis of CP‐I plasma baseline reported a mean baseline concentration of 0.685 nM [16]. The clinical studies used in that meta‐analysis included baseline data from different ethnicities and sexes, as well as studies where the *SLCO1B1* c.521T>C genotype was not identified. Therefore, this analysis was revised and expanded to collect the CP‐I baseline data where ethnicity, genotype, and sex were identified. Eight clinical studies reporting CP‐I plasma baseline concentrations in healthy adults were collected, forming the largest CP‐I clinical dataset to date. Data from 731 individual subjects and 18 datasets were considered for model verification (Table S1). These studies included CP‐I baseline data in different ethnicities (White vs. Japanese vs. Asian‐Indian), *SLCO1B1* c.521T>C genotypes (TT vs. TC vs. CC), and sexes (male vs. female). Only studies in which these covariates were clearly identified were included in the baseline analysis. In addition, CP‐I renal clearance data were collated from multiple clinical studies (Table S2).

To validate PBPK predictions of OATP1B‐mediated interactions, studies that reported CP‐I plasma concentrations by ethnicity and sex in the control phase were used. For DDI trials where the *SLCO1B1* genotype was not identified, it was assumed that the subjects carried the wild‐type allele (521TT). Plasma CP‐I concentrations following a range of rifampicin doses (150–600 mg), cyclosporine (20–100 mg), and probenecid (500–1000 mg) in diverse populations were collected from 21 datasets across nine clinical trials (Table S3). These inhibitors covered a range of inhibitory potency against OATP1B, with observed mean CP‐I AUCR ranging from 1.2 to 4.6 and *C*_max_ *R* of 1.4 to 6.7. All data analysis was conducted using Microsoft Excel 365 (Microsoft, Redmond, WA) and GraphPad Prism 10.2.2 (GraphPad software, La Jolla, CA). Reported pharmacokinetic profiles were digitized using WebPlotDigitizer version 4.5 (Automeris LLC, Frisco, Texas).

### 2.2 Coproporphyrin‐I PBPK Model Development

The reduced CP‐I PBPK model we previously published [19] was revised and implemented in Simcyp Simulator (V23; Certara, Sheffield, United Kingdom). The key updates in the model were the expression of the CL_active_ parameter as OATP1B1 and OATP1B3 CL_int_, and the CL_bile_ as MRP2‐mediated CL_int_ (details in Table 1). The relative contribution of OATP1B1 and OATP1B3 to hepatic uptake of CP‐I was based on the recent in vitro data [37]. Critical analysis of the other existing CP‐I models highlighted differences in model structure and inconsistency in the values of some of the input parameters, namely the fraction unbound in plasma (fu_plasma_), OATP1B1/3 intrinsic clearance (CL_int_), MRP2 CL_int_, renal excretion clearance, and CP‐I biosynthesis rates (*k*_syn_). An overview of these parameter values across different reported CP‐I PBPK models is shown in Table S4. To address the clinically reported sex differences in CP‐I baseline, the *k*_syn_ in females in the current model was set as 88% of the value in males based on reported differences in hemoglobin levels [19]. For the Japanese population, a 35% reduction in *k*_syn_ relative to White was needed to recover the CP‐I baseline in male 521TT subjects. The CP‐I PBPK model was evaluated for its predictive performance of CP‐I baseline and interactions with OATP1B inhibitors with different potency and in diverse populations.

**TABLE 1:** Input parameters for CP‐I PBPK model.

Input parameters (unit)		Values
Molecular weight (g/mol)		654.71^a^
LogP		2.53^a^
Compound type		Ampholyte^a^
pKa 1		3.56^a^
pKa 2		5.18^a^
B/P ratio		0.628^a^
Fraction unbound in plasma		0.069^b^
Distribution model		Full PBPK model
Prediction method		Method 2 (Rodgers and Rowland method)
CL_R_ (L/h)		2.3^c^
Biosynthesis rate (mg/day/kg)	White Asian‐Indian	M: 0.0036^d^, ^f^ F: 0.0032
	Japanese	M: 0.0024^e^, ^f^ F: 0.0021
**Permeability limited liver model**		
CL_pd_ (μL/min/10^6^ cells)		0.76^g^
OATP1B1 CL_int_ (μL/min/million cells)		106^h^ (RAF = 1)
OATP1B3 CL_int_ (μL/min/million cells)		31^h^ (RAF = 1)
MRP2 CL_int_ (μL/min/million cells)		0.612^i^ (RAF = 1)

Abbreviations: B/P, blood to plasma ratio; CL_int_, intrinsic transport clearance; CL_pd_, passive transport clearance; CL_R_, renal clearance; Mol, molecular; MRP2, multidrug resistance‐associated protein 2; OATP1B1/3, organic anion‐transporting polypeptide (OATP) 1B1/3; PBPK, physiologically based pharmacokinetics; p*K*a, acid dissociation constant.

^a^ Same value as Simcyp default “EB‐Coproporphyrin I” file.

^b^ Measured in protein binding assay [19].

^c^ Clinically observed weighted mean values of CP‐I CL_R_ (Table S2).

^d^ Estimated by Pop‐PK modeling [19].

^e^ Adjusted to recover the clinical baseline.

^f^ The sex ratio of *k*_syn_ was incorporated [19].

^g^ Measured in vitro as reported previously [19].

^h^ CL_active_ [19] was converted to OATP1B CL_int_. The reported contribution of OATP1B1 and OATP1B3 to hepatic uptake of CP‐I [37] was considered.

^i^ CL_bile_ [19] was converted to MRP2 CL_int_.

### 2.3 Prediction of CP‐I Baseline Considering Ethnicity/Genotype/Sex Effects

To account for differences in transporter activity across ethnicity, OATP1B1 abundance in Japanese wild‐type (521TT) was set at 58% of the value in White wild type, based on previous reports [7]. The effects of ethnicity were predicted using the Sim‐NEur Caucasian population file for White and the Sim‐Japanese population file for Asian‐Indian and Japanese, respectively (Figure S1). For predicting the effects of *SLCO1B1* c.521T>C genotype, default values for ET, IT, and PT populations were used for 521TT, TC, and CC, respectively. Demographic details (e.g., age range) were adjusted to match the reported clinical study, and simulations were run for at least 24 h to establish steady‐state concentrations (considered as CP‐I baseline).

### 2.4 Prediction of OATP1B‐Mediated CP‐I Interactions After the Administration of Weak to Strong OATP1B Inhibitors

Simcyp V23 default rifampicin and cyclosporine PBPK models were used (Tables S5 and S6) for prediction, with OATP1B1 and OATP1B3 *K*_*i*_ values for rifampicin (0.16 μM and 0.088 μM) and cyclosporine (0.019 and 0.032 μM [38]), respectively. The probenecid PBPK model previously developed and validated in our group [20] was adopted with minor modifications. To predict probenecid OATP1B‐mediated inhibition, in vitro IC_50_ values of 167 μM for OATP1B1 and 76 μM for OATP1B3, reported using CP‐I as a probe [39], were incorporated into the probenecid model (Table S7). The ability of the CP‐I PBPK model to predict dose‐dependent CP‐I interactions following the administration of rifampicin (150–600 mg), cyclosporine (20–100 mg), and probenecid (500–1000 mg) was investigated.

### 2.5 Estimation of In Vivo OATP1B *K*_*i*_ and Sensitivity Analyses

One proposed application of CP‐I PBPK models is to estimate OATP1B *K*_*i*_ for new investigational drugs using CP‐I clinical interaction data [14, 15]. The in vivo OATP1B *K*_*i*_ values for rifampicin, cyclosporine, and probenecid were estimated from CP‐I baseline and interaction data with the highest inhibitor dose and with the current CP‐I model. Subsequently, PBPK models of rosuvastatin and pitavastatin [40] (Tables S8 and S9) were used to assess the predictive performance of both in vitro and CP‐I‐estimated in vivo OATP1B *K*_*i*_ values (Table S10). Demographic data from rosuvastatin and pitavastatin DDI studies, and dosing regimens matched each clinical trial (Table S11).

A sensitivity analysis was performed to understand factors contributing to differences in estimated in vivo OATP1B1 *K*_*i*_. The effect of varying CP‐I fraction excreted unchanged in urine (fe) between 0% and 15% (and therefore varying contribution of hepatic elimination) on estimated OATP1B *K*_*i*_ was analyzed for (A) strong OATP1B inhibitor—rifampicin 600 mg, (B) moderate inhibitor—equivalent to cyclosporine low dose (100 mg), and (C) weak inhibitor—probenecid 1000 mg.

### 2.6 PBPK Model Evaluation

Predictions of CP‐I baseline were considered accurate if the ratio of predicted to observed value (*R*_pred/Obs_) fell within the 1.5‐fold error criterion (0.67 ≤ *R*_pred/Obs_ ≤ 1.5), as used in other biomarker PBPK model development [20]. Considering low AUCR for some of the cyclosporine and probenecid interactions, the evaluation of PBPK DDI predictive performance was done against more stringent acceptance criteria by Guest et al. [41], in addition to commonly used twofold criterion [42]. Analysis of prediction accuracy was also performed per weak, moderate, and strong OATP1B1 inhibition category, considering that a dose range was used for cyclosporine and rifampicin. Statistical comparison of predicted and observed PK parameters was performed by calculating the geometric mean fold error (GMFE) (Equation 1).

(1) $$\text{GMFE}=10^{x};x=\frac{\sum_{i=1}^{n}\left|\log_{10}\left(\frac{\text{predicted}\;\mathrm{PK}\;\text{paramete}r_{i}}{\text{observed}\;\mathrm{PK}\;\text{paramete}r_{i}}\right)\;\right|}{n}$$

where PK parameter_*i*_ refers to CP‐I baseline, *C*_max_ *R* or AUCR value, *n* = number of studies.

### 2.7 Study Design and Appropriate Exposure Metrics for Monitoring CP‐I in Phase I Study

To facilitate the design of prospective clinical trials with OATP1B inhibitors in early clinical stages, further consideration was given to the appropriate exposure metrics (AUCR or *C*_max_ *R*). Four virtual OATP1B inhibitors with different potencies ranging from weak to strong OATP1B, with either short or long *t*_1/2_, were investigated (Table S12); simulation details are provided in Appendix S1.

## 3 Results

### 3.1 Prediction of CP‐I Baseline in Different Ethnicities, Genotypes, and Sex

**FIGURE 2:**
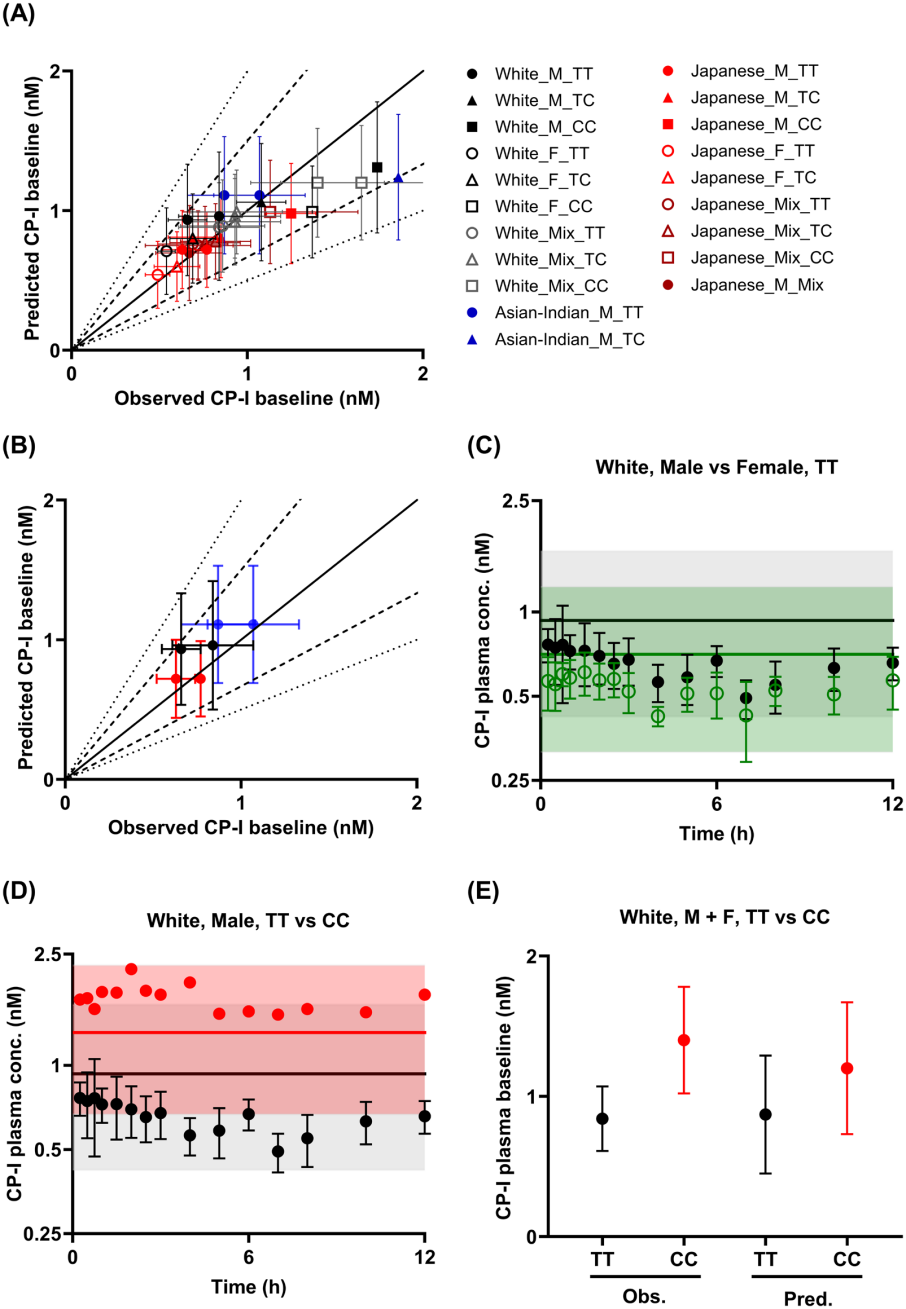
Observed and predicted CP‐I plasma baseline in different ethnicities, *SLCO1B1* c.521T>C genotypes, and male versus female populations using the harmonized CP‐I PBPK model. (A) All scenarios (B) Ethnicity effect on CP‐I baseline. The observed plasma baseline data from White [3], Asian‐Indian [22, 23], and Japanese [24, 26] male TT populations are shown. (A, B) Colors (black, blue, and red) represent different ethnicities (White, Asian‐Indian, and Japanese), whereas shapes (circle, triangle, and square) indicate *SLCO1B1* c.521T>C genotypes (TT, TC, and CC), respectively. Mix represents clinical studies where the ethnicity, genotype, and sex of the subjects were identified, but the baseline data were not reported separately by genotype or sex. Filled or unfilled shapes represent sex differences (male and female). Symbols are presented as mean ± standard deviation. Solid, dashed and dotted lines on the graphs represent the line of unity, 1.5‐fold, and twofold error criteria, respectively. (C) Predicted sex effect on CP‐I baseline. The individual observed plasma concentrations over time are from White male and female TT subjects [3]. (D) Predicted effect of *SLCO1B1* c.521T>C on CP‐I baseline. The individual observed plasma concentrations over time are from White male TT or CC subjects [3]. (C, D) The predicted average plasma concentrations over time are depicted with solid lines (mean) and shaded areas (5th and 95th percentiles). Symbols represent observed mean baseline levels ± standard deviation. (E) The predicted CP‐I baseline in 521TT and 521CC White individuals and comparison to reported data from a large‐scale clinical study (521TT, *n* = 116 and 521CC, *n* = 11) [28].

CP‐I baseline in 18 scenarios, including different ethnicities (White vs. Japanese vs. Asian‐Indian), *SLCO1B1* c.521T>C genotypes (TT vs. TC vs. CC), and sex (male vs. female), was predicted and evaluated against reported CP‐I baseline from 16 datasets. CP‐I PBPK model successfully predicted CP‐I baseline plasma concentrations, with 97% of predicted values within 1.5‐fold of the observed data (Figure 2A, GMFE = 1.1, Table S13). The predicted CP‐I baseline in different ethnic groups in 521TT individuals is shown in Figure 2B. The individual effect of either sex or genotype on the CP‐I baseline over 24 h in White individuals is shown in Figure 2C,D, respectively. The predicted ratio of CP‐I baseline concentrations in CC relative to TT in mixed male and female White subjects of 1.4 (Figure 2E, Table S13) was within < 20% error relative to the reported ratio of 1.6 in the large‐scale clinical study [28]. The predicted baseline ratio of females to males in White was 0.8 (Table S13), in agreement with the observed ratio of 0.8 [3].

The clinically observed values of CP‐I CL_R_ ranged from 1.1 to 3.0 L/h, and a weighted mean of 2.3 L/h was implemented in our model (Table S2). As such, the contribution of renal elimination (fe) of CP‐I in the current model was 10%, consistent with previous clinical reports [33]. In contrast, some of the existing CP‐I PBPK models assume minimal renal elimination of CP‐I (e.g., 3% in the Simcyp “EB‐Coproporphyrin‐I” default model, Table S4). Fractions transported via OATP1B1 (fT_OATP1B1_) and OATP1B3 (fT_OATP1B3_) were 66% and 24%, respectively. The model assumed minimal contribution of passive diffusion to CP‐I hepatic uptake (< 1%).

### 3.2 Prediction of OATP1B‐Mediated CP‐I Interactions Using In Vitro *K*_*i*_ Values for Weak to Strong OATP1B Inhibitors

**FIGURE 3:**
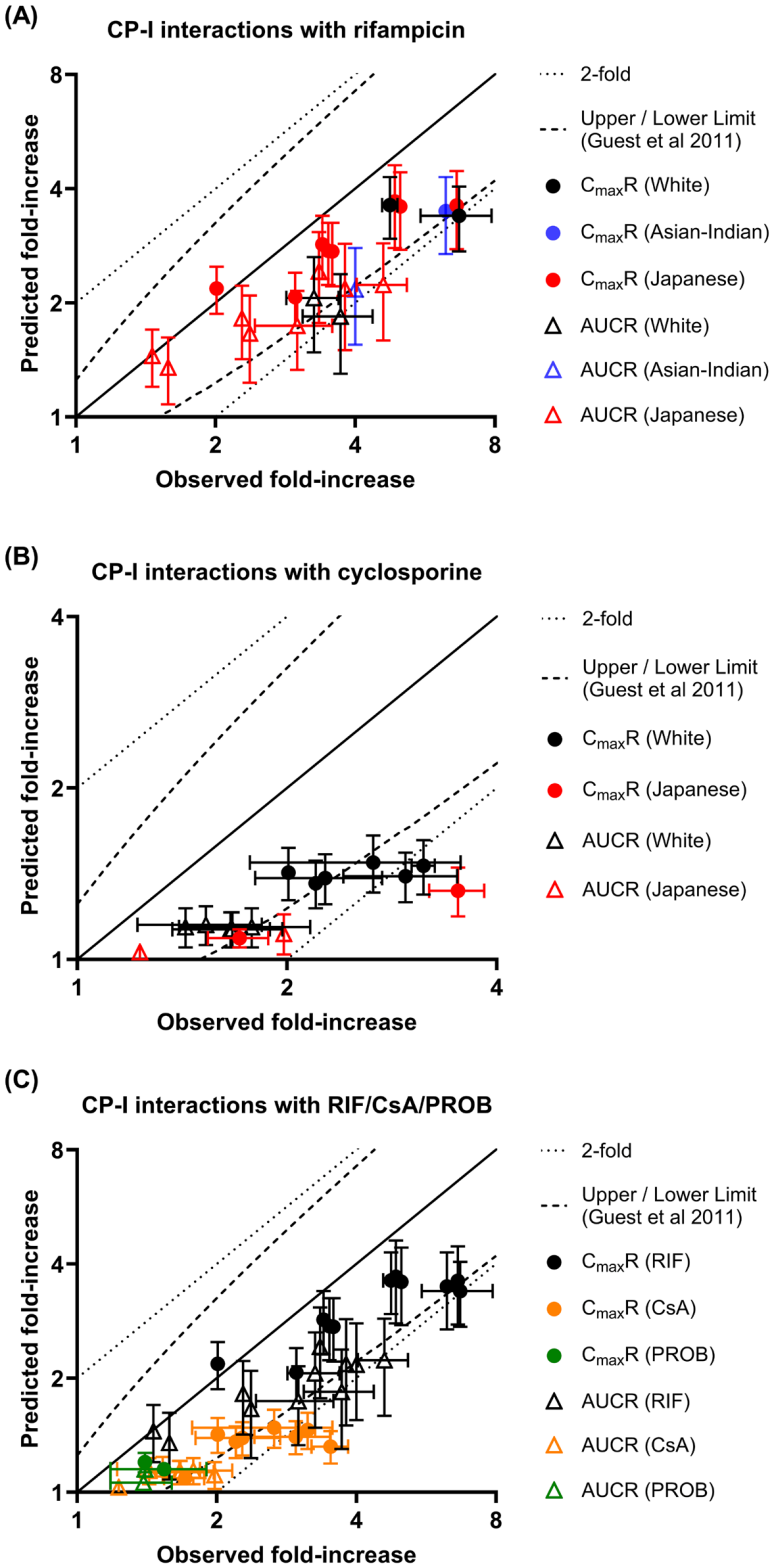
Comparison of observed and predicted changes in CP‐I *C*_max_ *R* and AUCR in the presence of prototypical OATP1B inhibitors, rifampicin, cyclosporine, and probenecid. Predictions were made with in vitro OATP1B *K*_*i*_ values implemented in inhibitor models and using the harmonized CP‐I PBPK model proposed here. (A) Rifampicin interactions (B) cyclosporine interactions, and (C) interactions with all three inhibitors, rifampicin, cyclosporine, and probenecid. (A, B) Colors (black, red, and blue) indicate different ethnicities (White, Japanese, and Asian‐Indian), respectively. (C) Colors (black, orange, and green) represent OATP1B inhibitors (rifampicin, cyclosporine, and probenecid). Circles and triangles represent CP‐I *C*_max_ *R* and AUCR, respectively. Symbols are presented as mean ± standard deviation. Solid, dashed, and dotted lines on the graphs represent the line of unity, Guest acceptance criterion, and 2‐fold error, respectively.

**FIGURE 4:**
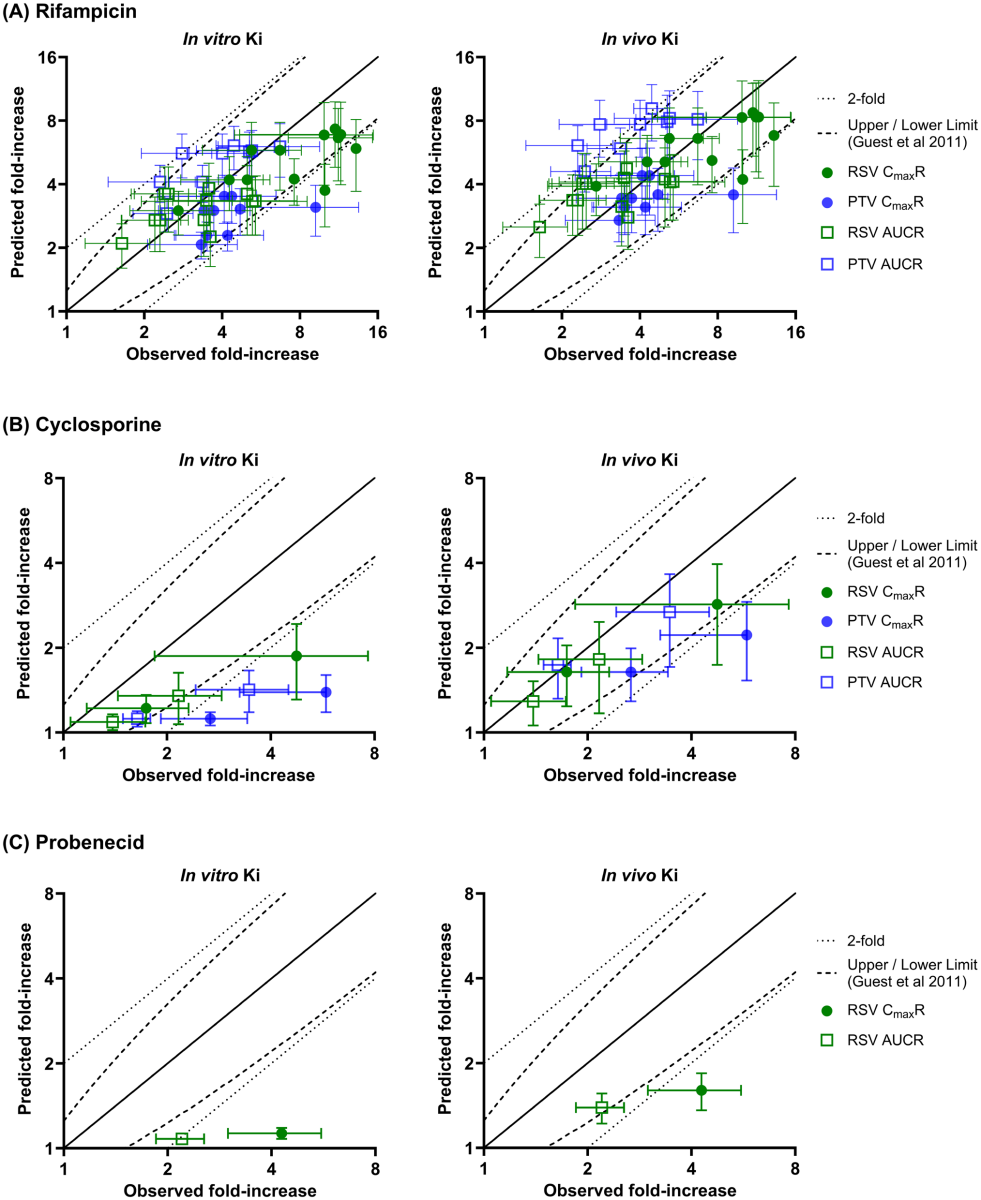
Observed and predicted changes in rosuvastatin/pitavastatin *C*_max_ *R* and AUCR after the administration of (A) rifampicin (150–600 mg), (B) cyclosporine (20–75 mg), or (C) probenecid (1000 mg) using in vitro or in vivo *K*_*i*_ estimated from CP‐I interaction data. Blue and green symbols represent pitavastatin and rosuvastatin, respectively. Circles and squares represent *C*_max_ *R* and AUCR, respectively. Symbols are presented as mean ± standard deviation. Solid, dashed, and dotted lines represent the line of unity, Guest acceptance criterion [41], and 2‐fold error, respectively. In the case of rosuvastatin, the model implements total renal clearance (CL_R_) instead of OAT1/3 intrinsic clearance (OAT1/3 CL_int_) and therefore the inhibitory effect of probenecid on OAT1/3 was not explicitly accounted for. This is a likely contributing factor to the underprediction of rosuvastatin *C*_max_ *R* and AUCR in the presence of probenecid, even when the in vivo *K*_*i*_ value was used.

Dose‐dependent interactions with CP‐I following an oral dose range of rifampicin (150–600 mg), cyclosporine (20–100 mg), and probenecid (500–1000 mg) were predicted in diverse populations and compared with observed CP‐I interaction profiles from 21 datasets. The model predicted 76% of CP‐I *C*_max_ *R* and AUCR of the overall dataset within the Guest criterion (GMFE = 1.5). In the case of rifampicin interactions, 82% of predicted *C*_max_ *R* and AUCR were within the Guest acceptance criterion (GMFE = 1.5), but an underprediction tendency was noted for the highest rifampicin dose (Figure 3A, Table S14). The extent of interaction with cyclosporine using in vitro *K*_*i*_ was consistently underpredicted on average by 1.6‐fold, with 63% of *C*_max_ *R* and AUCR predicted within the Guest criterion (Figure 3B, Table S14). An underprediction trend was also evident in the case of probenecid‐CP‐I interactions, although predicted *C*_max_ *R* and AUCR were within the Guest criterion (Figure 3C, Table S14). Similar prediction trends, in particular underprediction of cyclosporine and probenecid DDIs, were also apparent when in vitro *K*_*i*_ values were used to predict rifampicin, cyclosporine, and probenecid DDIs with representative OATP1B probes, rosuvastatin and pitavastatin (Figure 4).

### 3.3 Estimation of In Vivo OATP1B *K*_*i*_ With CP‐I PBPK Model and Sensitivity Analyses

From the DDI perspective, an important application of modeling CP‐I data is to estimate an in vivo OATP1B *K*_*i*_ for a new inhibitor. This value can inform subsequent DDI predictions for OATP1B substrates, aiming to overcome the commonly observed underprediction of these DDIs when using in vitro *K*_*i*_ values. The in vivo OATP1B1 *K*_*i*_ estimated by the harmonized CP‐I PBPK model from CPI‐interaction data with the highest doses of rifampicin, cyclosporine, and probenecid are summarized in Table S10. Overall, the in vivo *K*_*i*_ values were 3–9 fold lower than in vitro values, noted for rifampicin and cyclosporine, respectively.

Subsequent application of CPI‐informed in vivo *K*_*i*_ improved the prediction accuracy of interactions with rosuvastatin/pitavastatin. The most prominent improvement was noted for cyclosporine and probenecid; in the case of cyclosporine, 88% of pitavastatin/rosuvastatin *C*_max_ *R* and AUCR values were predicted within the Guest criterion (relative to 50% when in vitro *K*_*i*_ was used) (Figure 4B). Similarly, the use of in vivo *K*_*i*_ resulted in 50% of probenecid‐rosuvastatin DDIs predicted within the error margin compared to 0% when in vitro *K*_*i*_ was used (Figure 4C). Prediction accuracy of rifampicin‐pitavastatin/rosuvastatin DDIs was comparable to the use of in vitro *K*_*i*_ (Figure 4A).

Sensitivity analysis showed that the differences in % contribution of renal and hepatic elimination between published CP‐I models can explain differences in the estimated OATP1B *K*_*i*_ for investigational inhibitors with differing inhibitory potencies. The estimated in vivo OATP1B1 *K*_*i*_ for a strong inhibitor (equivalent to rifampicin 600 mg) is sensitive to the assumed % contribution of the parallel pathway (renal elimination) and fT_OATP1B1_ in CP‐I models. An increase in fe from 0% to 15% (fT_OATP1B1_ change from 73% to 62%) resulted in a sevenfold decrease in the estimated OATP1B1 *K*_*i*_ and subsequently a 37% difference in predicted rosuvastatin AUCR (AUCR from 3.6 to 4.9; Figure S2A). In the case of a moderate inhibitor (low dose of cyclosporine), a twofold difference in OATP1B1 *K*_*i*_ was noted depending on the assumed contribution of hepatic versus renal in the CP‐I model, causing only a 16% difference in predicted rosuvastatin AUCR (Figure S2B). For interactions with a weak inhibitor (probenecid), the effect was minor, resulting in a 6% difference in predicted rosuvastatin AUCR (Figure S2C).

### 3.4 Study Design and Appropriate Exposure Metrics for Monitoring CP‐I in Phase I Study

**FIGURE 5:**
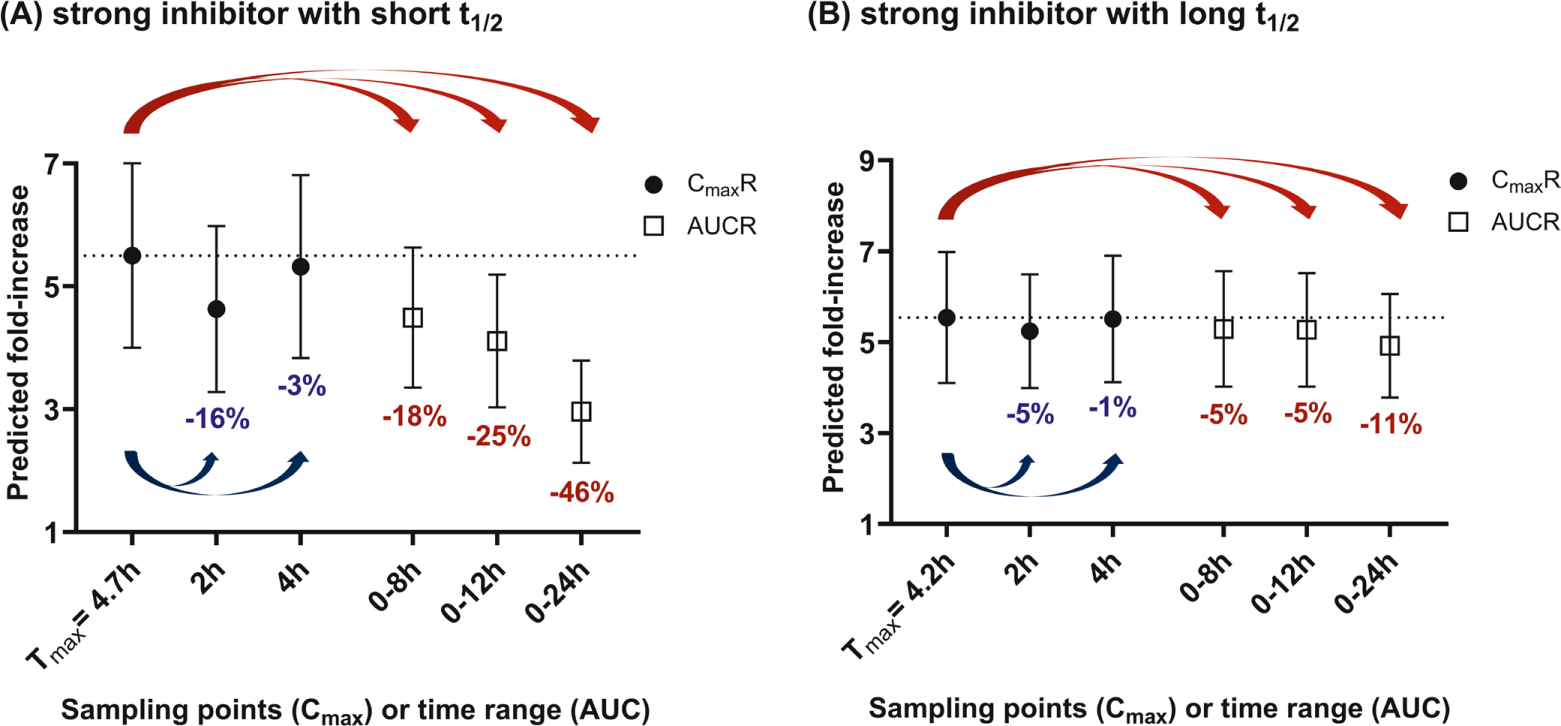
Predicted CP‐I *C*_max_ *R* and AUCR at steady‐state when virtual OATP1B inhibitors are administered once daily. (A) strong inhibitor with short *t*_1/2_ or (B) strong inhibitor with long *t*_1/2_. Circles and squares represent CP‐I *C*_max_ *R* and AUCR, respectively. Symbols represent predicted mean ± standard deviation. The values below the symbols indicate the percent difference from simulated CP‐I *C*_max_ *R* (*T*_max_).

CP‐I plasma concentration time‐profiles were simulated following multiple doses of virtual inhibitors with different inhibitory potencies against OATP1B and PK relative to CP‐I. Simulated steady‐state CP‐I profiles, *C*_max_ *R*, and AUCR after once‐daily administration of strong inhibitors with either a short or long *t*_1/2_ are illustrated in Figure 5. For the strong inhibitor with a short *t*_1/2_, the use of *C*_max_ *R* predicted a higher magnitude of DDI (46% higher than AUCR_0–24h_). Deviations of sampling points from the simulated *T*_max_ of 4.7 h had an effect on the estimated *C*_max_ *R*; it decreased by 3% at a time point 0.7 h before *T*_max_ (*T* = 4 h), and by 16% at *T* = 2 h (Figure 5A). For the moderate inhibitor with a short *t*_1/2_, similar findings were obtained, as *C*_max_ *R* was more sensitive to interactions than AUCR (Figure S3A).

In the case of a strong inhibitor with a long *t*_1/2_, the difference in the magnitude of interaction between the use of *C*_max_ *R* and AUCR was only 11%. Deviations from *T*_max_ had less of an effect (< 10%, Figure 5B) on calculating *C*_max_ *R*. A weak OATP1B inhibitor with a short *t*_1/2_ exhibited similar trends to a strong inhibitor with a long *t*_1/2_, where both *C*_max_ *R* and AUCR resulted in comparable estimates of the magnitude of CP‐I interaction (Figure S3B).

## 4 Discussion

### 4.1 Prediction of CP‐I Baseline in Diverse Populations

This study developed a harmonized CP‐I PBPK model and critically evaluated the ability of this model to predict the effects of different ethnicities, *SLCO1B1* c.521T>C genotypes, and sex on CP‐I baseline and the magnitude of OATP1B‐mediated DDIs. The robustness of the CP‐I PBPK model was validated against the largest CP‐I baseline dataset to date, consisting of 731 subjects with identified ethnicity, genotype, and sex. Key differences to other published models were noted in the revised value of CP‐I CL_R_ and therefore the contribution of renal elimination of 10%, in contrast to some of the existing models that assume minimal contribution of the parallel elimination pathway (Table S4). As a consequence, the fT_OATP1B1_ of CP‐I of 66% in the current model is slightly lower compared to other models (e.g., 71% in the Simcyp default model) leading to a lower predicted magnitude of OATP1B‐mediated interactions of CP‐I. In contrast to other CP‐I models (Table S4), the harmonized model implements differences in CP‐I synthesis between male and female, in addition to per weight basis, providing the ability to simulate diverse populations in a more robust way.

### 4.2 Prediction of OATP1B‐Mediated CP‐I Interactions

Ability of the CP‐I PBPK model to predict CP‐I interactions with rifampicin, cyclosporine, and probenecid was evaluated against a total of 21 clinical observations. Interactions across the 150–600 mg dose range of rifampicin were generally predicted well (82% within the error margins, Figure 3A). Rifampicin interactions also included cases of weak and moderate OATP1B1 inhibition, which contributed to comparable performances of CP‐I *C*_max_ *R* for cases of weak (83%) and moderate (79%) OATP1B1 inhibition, despite underprediction noted for cyclosporine and probenecid studies in these categories. The underprediction of cyclosporine and probenecid CP‐I interactions when in vitro OATP1B *K*_*i*_ values were used in inhibitor PBPK models was not unique to our harmonized CP‐I PBPK model; similar trends were noted for predictions by the Simcyp CP‐I default model (Figure S4). This underprediction of OATP1B interactions was also evident for DDIs with the prototypical OATP1B probes, rosuvastatin and pitavastatin (Figure 4B,C, Figure S5). These findings are consistent with previous reports [43, 44], and are likely attributed to discrepancies between in vitro and in vivo OATP1B *K*_*i*_ values. The in vivo OATP1B *K*_*i*_ values estimated for inhibitors investigated here were up to ninefold lower than the in vitro values and within the range of previously reported data based on fitting of either PBPK or popPK models to CP‐I interaction data (Table S10).

Sensitivity analyses highlighted important considerations when using the CP‐I model for estimation of in vivo *K*_*i*_ for novel OATP1B inhibitors. Models that assume that CP‐I is almost exclusively eliminated via the hepatic route (e.g., 97% assumed in the Simcyp default model, Table S4) estimate higher in vivo *K*_*i*_ compared to the harmonized model, which assumes a 10% contribution of renal excretion. This is not surprising, as a higher contribution of the parallel/non‐inhibited pathway (i.e., renal excretion) leads to lower fT_OATP1B1_ for CP‐I and, consequently, lower estimates of the in vivo *K*_*i*_ of OATP1B inhibitors. The in vivo *K*_*i*_ values estimated by our current CP‐I model are seen as more conservative and therefore provide the worst‐case scenario for DDI risk assessment of novel inhibitors. Sensitivity of estimated in vivo *K*_*i*_ for any new inhibitor to the assigned contributions of hepatic and parallel elimination pathways in the biomarker model needs to be considered when using EB biomarker models for such purposes.

### 4.3 Study Design and Appropriate Exposure Metrics for Monitoring CP‐I in Phase I Study

To support the design of prospective clinical trials for the risk assessment of OATP1B‐mediated DDIs, virtual OATP1B inhibitors with different *K*_*i*_ (weak to strong) and *t*_1/2_ (short to long) were created, and the appropriate exposure metrics for monitoring CP‐I were evaluated. These investigations aimed to enhance the efficiency of phase I trials by evaluating conditions that reduce unnecessary resources (e.g., sampling points) while obtaining sufficient information for DDI risk assessment. For the strong and moderate inhibitors with short *t*_1/2_, CP‐I *C*_max_ *R* gave a higher estimate of DDI risk, whereas the use of CP‐I AUCR could lead to underestimation of DDI effect and misclassification of inhibitory potential. For the weak inhibitor with short *t*_1/2_ and the strong inhibitor with long *t*_1/2_, AUCR was as sensitive as *C*_max_ *R* (Figure 5 and Figure S3). Considerations and appropriate exposure metrics for monitoring CP‐I in phase I study are summarized in Table 2. Complete capture of the maximal extent of DDI can be achieved through single dose assessment when the inhibitor has a short *t*_1/2_, but repeated doses are required for inhibitors with a long *t*_1/2_.

**TABLE 2:** Summary of considerations and appropriate exposure metrics for monitoring CP‐I in phase I study.

Virtual OATP1B inhibitor	Number of doses required to capture the maximum inhibition	Appropriate exposure metrics	Considerations
(a) Strong inhibitor with short *t*_1/2_	Single dose	*C*_max_ *R*	*C*_max_ *R* gives a higher estimate of DDI risk Using AUCR rather than *C*_max_ *R* could lead to underestimation of DDI effect and misclassification of inhibitory potential Deviation in sampling points from *T*_max_ has an effect on the calculation of *C*_max_ *R* For accurate calculation of CP‐I *C*_max_ *R*, several sampling points around CP‐I *T*_max_ should ideally be used to account for interindividual variability
(b) Moderate inhibitor with short *t*_1/2_	Single dose		
(c) Weak inhibitor with short *t*_1/2_	Single dose	*C*_max_ *R* and AUCR	AUCR is as sensitive as *C*_max_ *R* Deviation in sampling points from *T*_max_ has less effect on the calculation of *C*_max_ *R*
(d) Strong inhibitor with long *t*_1/2_	Multiple dose		

In the case of strong and moderate inhibitors with short *t*_1/2_, the deviation in sampling points from *T*_max_ has a substantial impact on the calculation of *C*_max_ *R*. Since CP‐I *T*_max_ depends on the kinetics of the inhibitor, interindividual variability in *T*_max_ needs to be considered when using a reduced number of sampling points (e.g., 1 or 2 points). For accurate calculation of CP‐I *C*_max_ *R*, several sampling points around CP‐I *T*_max_ should ideally be used to account for interindividual variability. In the case of a weak inhibitor with a short *t*_1/2_ or a strong inhibitor with a long *t*_1/2_, the deviation in sampling points from *T*_max_ has less effect on calculating *C*_max_ *R*. Overall, the *C*_max_ *R* was a more sensitive metric than AUCR, and accurately estimating CP‐I *C*_max_ *R* should take priority over capturing the complete AUC. These detailed investigations align with previous discussions by the ITC and IQ Consortium on the interpretation of systemic biomarker exposure in relation to the half‐life (*t*_1/2_) of inhibitors [14, 16].

In summary, the current study critically assessed existing CP‐I PBPK models and developed a harmonized CP‐I PBPK model. Its robustness was confirmed against the largest CP‐I baseline dataset to date, and its ability to predict OATP1B‐mediated interactions with weak to strong OATP1B inhibitors in diverse populations. The biomarker‐informed PBPK modeling approach aims to utilize CP‐I data to estimate in vivo OATP1B *K*_*i*_, with the aim of improving the accuracy of subsequent DDI predictions with OATP1B substrates. Differences in the assumed contribution of hepatic and renal elimination in CP‐I models led to differences in estimated OATP1B *K*_*i*_ for the same OATP1B inhibitors; a higher contribution of the parallel elimination pathway/renal excretion in CP‐I models resulted in a lower estimated in vivo *K*_*i*_ of inhibitors for the same AUCR. Given the uncertainty/range in observed CL_R_ to inform the model, the assumption of higher fe is recommended when using the biomarker model to estimate inhibitor in vivo *K*_*i*_ to obtain a conservative prediction of DDI risk. The study investigated appropriate exposure metrics for monitoring CP‐I and highlighted the advantages of using *C*_max_ *R* in the case of strong and moderate inhibitors with short *t*_1/2_. *C*_max_ *R* and AUCR were comparable for the assessment of DDI risk with strong OATP1B inhibitors with long *t*_1/2_/weak inhibitors with short *t*_1/2_. The findings of this study inform the design of prospective clinical trials and recommend the use of *C*_max_ *R* as a more sensitive metric for the risk assessment of OATP1B‐mediated CP‐I–drug interactions.

## Author Contributions

Y.U., S.P.F.T., D.S., and A.G. wrote the manuscript; Y.U., S.P.F.T., D.S., and A.G. designed the research. Y.U. and S.P.F.T. performed the research.

## Conflicts of Interest

The authors declare no conflicts of interest.

## Supporting information


Appendix S1.
