# Supplementary material for: Genotype, Ethnicity, and Drug–Drug Interaction Modeling as Means of Verifying Transporter Biomarker PBPK Model: The Coproporphyrin‐I Story
Source: CPT Pharmacometrics Syst Pharmacol. 2025 Mar 10;14(5):941–53. doi: 10.1002/psp4.70008 (PMC12072222; doi:10.1002/psp4.70008)
Supplement: Supplementary file 1 — Appendix S1. [file PSP4-14-941-s001.pdf]

Supplementary Material

Genotype, ethnicity and drug-drug interaction modelling as means of verifying transporter biomarker PBPK model: The coproporphyrin-I story

Yuki Ujihira[1,2], Shawn Pei Feng Tan[1], Daniel Scotcher[1] and Aleksandra Galetin[1]

[1] Centre for Applied Pharmacokinetic Research, School of Heath Sciences, University of Manchester, Manchester, UK

[2] Laboratory for Safety Assessment and ADME, Pharmaceuticals Research Center, Asahi Kasei Pharma Corporation, Shizuoka, Japan.

The covariates evaluated in this study

The current study focused on the effect of ethnicity, *SLCO1B1* c.521T>C genotype, and sex on CP-I baseline and OATP1B-mediated interaction due to availability of clinical data. Other possible covariates (e.g., body weight, height) were not assessed due to the lack of available clinical data for validation.

Prediction of CP-I baseline considering ethnicity/genotype/sex effects

There were no reports on the abundance of OATP1B in Asian-Indian populations. In Simcyp simulator, Japanese and Chinese populations are available for Asian population. It has been reported that rosuvastatin AUC increased by 79%, 66%, and 26% in Chinese, Japanese, and Asian-Indian, respectively, compared to White, due to ethnic differences in OATP1B transporter activity.[1] Therefore, the Japanese population, which has reported variations in OATP1B activity closer to those observed in the Asian-Indian population, was used as a surrogate.

To account for differences in transporter activity across ethnicity, OATP1B1 abundance in Japanese wild-type (521TT) was set at 58% of that in White wild-type, based on previous reports.[2] The effects of ethnicity were predicted using Sim-NEurCaucasian population file for White and Sim-Japanese population file for Asian-Indian and Japanese, respectively (Figure S1). For predicting the effects of *SLCO1B1* c.521T>C genotype, default values for ET, IT, and PT populations were used for 521TT, TC, and CC, respectively.

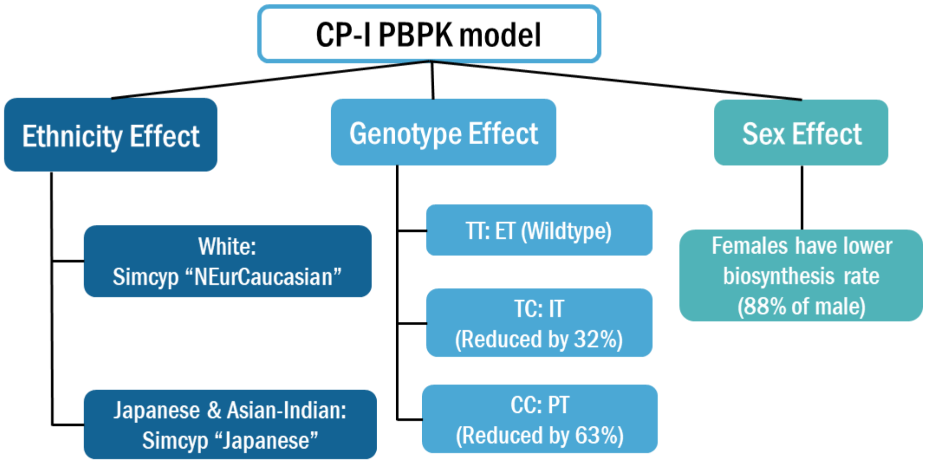

Figure S1. Simulation conditions in the Simcyp simulator to predict CP-I plasma baseline in diverse populations

TT, TC, and CC represent the *SLCO1B1* c.521T>C genotypes in clinical studies. ET, IT, and PT indicate extensive, intermediate, and poor transporting phenotypes of the OATP1B1 transporter in Simcyp population, respectively.

Differences in parameters between the CP-I PBPK model developed in this study and those published in other studies

The CP-I PBPK model developed in this study was compared with other CP-I PBPK models published in the literature (Table S4). The value of $f_{u\ plasma}$ incorporated in the current CP-I model is based on our previous report.[3] In this study, $f_{u\ plasma}$ was measured using labelled CP-I after 5 hours to allow equilibrium of protein binding; in addition, nonspecific binding, stability, and recovery rate were calculated. The $f_{u\ plasma}$ incorporated in the Simcyp CP-I model was measured using a rapid equilibrium dialysis method; however, details of experimental procedures were not available.[4]

The current study collated clinical data on renal elimination of CP-I considering inconsistency in the literature (Table S2). While Gu et al.[5] cited the study that reported approximately 70-80% of CP-I is excreted in bile and the remainder in urine,[6] Kimoto et al.[7] cited the study showing 3% urinary elimination of 14C-labeled CP-I.[8] Other modelling studies reported higher contribution of CP-I renal elimination, e.g. Yoshida et al.[9] reported 13% and Barnet et al.[10] of approximately 15%.

It has been reported that the contribution of glomerular filtration clearance to CP-I CL_R was 63%.[11] CP-I is known to be a substrate of multidrug resistance protein MRP2 and MRP3, and possibly OATP4C1[12] transporters, suggesting that some active renal secretion may occur. At the time this study was conducted, there was insufficient *in vitro* and clinical DDI data on the involvement of renal transporters in CP-I elimination to support the consideration of active renal transport in the PBPK model analyses.

Sensitivity analysis

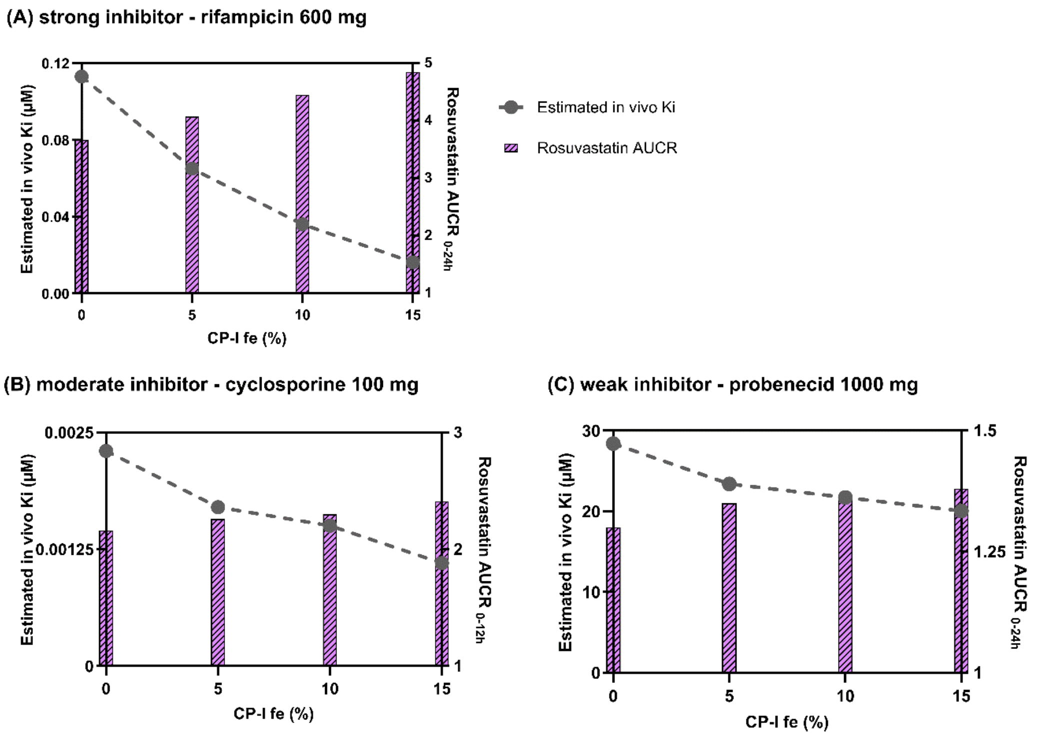

Figure S2. Sensitivity analysis into impact of CP-I fe and therefore varying contribution of hepatic elimination ($fT_{OATP1B1}$) in CP-I PBPK model on estimated *in vivo* OATP1B1 Ki of three inhibitors and subsequent DDIs with rosuvastatin. (A) Strong inhibitor – rifampicin 600 mg (B) Moderate inhibitor – cyclosporine 100 mg (low dose) (C) Weak inhibitor – probenecid 1000 mg X-axis represents the proportion of CP-I fe. Left Y-axis and black symbols show the model estimated *in vivo* OATP1B1 Ki of inhibitors after the administration of rifampicin 600 mg, cyclosporine 100 mg or probenecid 1000 mg. Right Y-axis and pink bar represent the predicted rosuvastatin AUCR after the administration of same doses of rifampicin, cyclosporine, or probenecid with estimated *in vivo* OATP1B Ki. An increase in fe from 0% to 15% ($fT_{OATP1B1}$ change from 73% to 62%) resulted in (A) 7-fold reduction in estimated OATP1B1 Ki and 37% difference in predicted rosuvastatin AUCR (i.e., AUCR 3.6 to 4.9), (B) 2-fold reduction in OATP1B1 Ki, causing 16% difference in predicted rosuvastatin AUCR (i.e., AUCR 2.1 to 2.4), (C) 1.4-fold reduction, resulting in 6% difference in predicted rosuvastatin AUCR (i.e., AUCR 1.3 to 1.4). fe, fraction excreted unchanged in urine; $fT_{OATP1B1}$, fraction transported via OATP1B1 transporter.

Study design and appropriate exposure metrics for monitoring CP-I in phase I study

CP-I PBPK model was combined with four virtual OATP1B inhibitors with different potencies ranging from weak to strong OATP1B, with either short or long $t_{1/2}$ (Table S12). Virtual inhibitors that resulted in predicted changes in CP-I C_{max} or AUC of greater than 5-fold, between 2- to 5-fold, 1.25- to 2-fold were defined as strong, moderate, and weak inhibitors, respectively. Inhibitors with $t_{1/2}$ of ≤ 5h were defined as short $t_{1/2}$, and those with $t_{1/2}$ of ≥ 20h were defined as long $t_{1/2}$ inhibitors. Simulated CP-I PK profile, C_{max}R, and AUCR following multiple doses of OATP1B inhibitors were compared.

Steady-state CP-I C_{max}R and AUCR after once-daily administration of moderate or weak inhibitors with short $t_{1/2}$ are illustrated in Figure S3. For the moderate inhibitor with short $t_{1/2}$, the value of $AUCR_{0\text{-}24h}$ was 45% lower compared to C_{max}R. For the weak inhibitor with short $t_{1/2}$, $AUCR_{0\text{-}24h}$ was 18% lower compared with C_{max}R. The moderate inhibitor with short $t_{1/2}$ showed high sensitivity to C_{max}R, similar to strong inhibitor with short $t_{1/2}$. In contrast, the weak inhibitor with short $t_{1/2}$ exhibited similar trends to strong inhibitors with long $t_{1/2}$, where monitoring of both CP-I C_{max}R and AUCR resulted in comparable estimate of the magnitude of interaction.

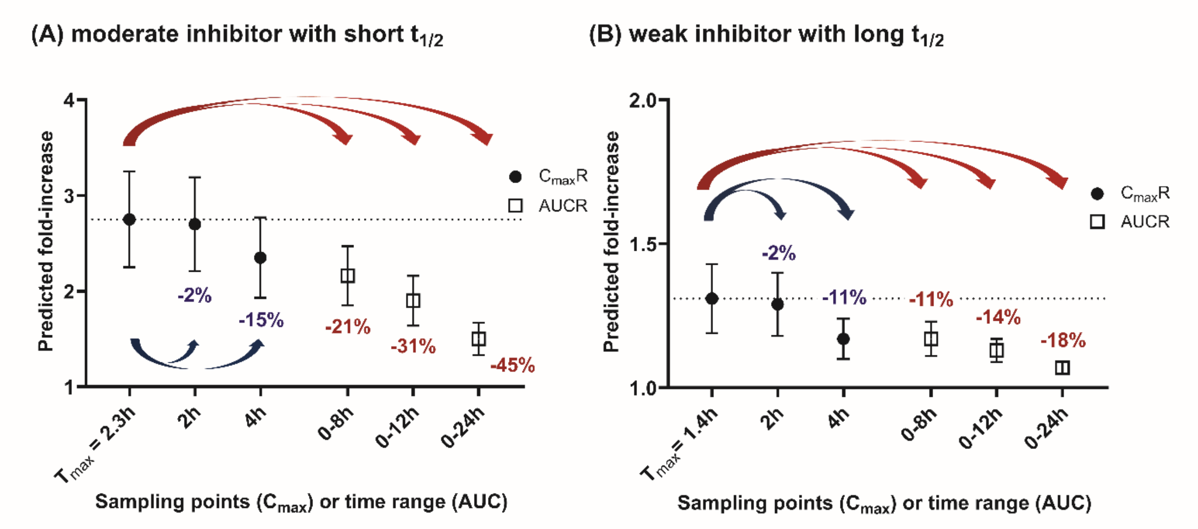

Figure S3. Predicted CP-I C_{max}R and AUCR at steady state when virtual OATP1B inhibitors are administered once daily. (A) Moderate inhibitor with short $t_{1/2}$ (B) Weak inhibitor with long $t_{1/2}$

For the moderate inhibitor with short $t_{1/2}$, the value of $AUCR_{0\text{-}24h}$ was 45% lower compared to C_{max}R. For the weak inhibitor with short $t_{1/2}$, $AUCR_{0\text{-}24h}$ was 18% lower compared with C_{max}R. The moderate inhibitor with short $t_{1/2}$ showed high sensitivity to C_{max}R, similar to strong inhibitor with short $t_{1/2}$. In contrast, the weak inhibitor with short $t_{1/2}$ exhibited similar trends to strong inhibitors with long $t_{1/2}$, where monitoring of both CP-I C_{max}R and AUCR resulted in comparable estimate of the magnitude of interaction. Circles and squares show CP-I C_{max}R and AUCR, respectively. Symbols represent predicted mean ± standard deviation. The values below the symbols indicate the rate of reduction from C_{max}R (T_{max}).

Prediction of CP-I baseline and OATP1B-mediated CP-I interactions using Simcyp default CP-I PBPK model

Simcyp default CP-I model predicted 61% of the values to be within 1.5-fold of the observed data, (Figure S4A), compared to 97% by the harmonized model. Prediction of OATP1B-mediated interactions exhibited comparable trends to the CP-I model developed in this study, with good predictive performance of rifampicin interactions, and under-prediction of interactions with cyclosporine and probenecid (Figure S4B).

(A)

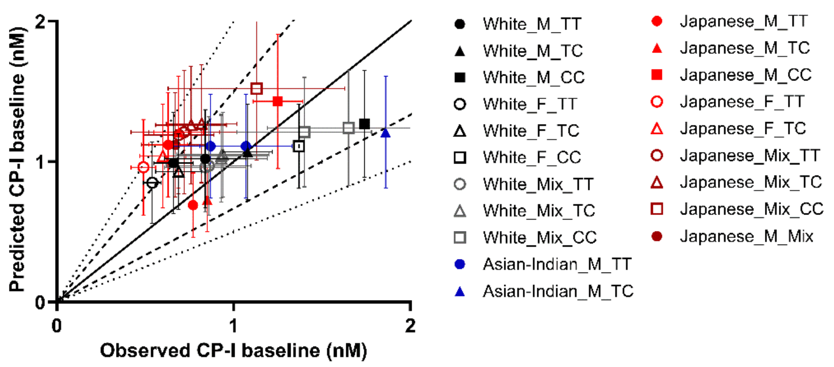

(B)

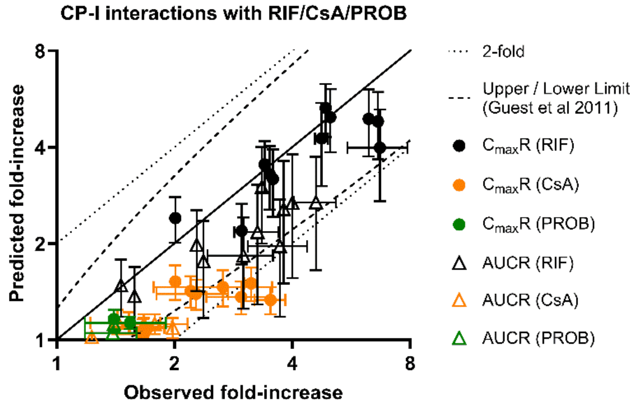

Figure S4. Observed and predicted CP-I plasma baseline and OATP1B-mediated CP-I interactions in the presence of prototypical OATP1B inhibitors, using the Simcyp default CP-I PBPK model.

(A) Observed and predicted CP-I plasma baseline in different ethnicities, *SLCO1B1* c.521 T>C genotypes, and male vs. female populations. Colours (black, blue, red) represent different ethnicities (White, Asian-Indian, Japanese), while shapes (circle, triangle, square) indicate *SLCO1B1* c.521 T>C genotypes (TT, TC, CC), respectively. Filled or unfilled shapes represent gender differences (male, female). Symbols are presented as mean ± standard deviation. Solid, dashed and dotted lines on the graphs represent the line of unity, 1.5-fold, and 2-fold error criteria, respectively.

(B) Comparison of observed and predicted changes in CP-I $C_{max}R$ and AUCR in the presence of prototypical OATP1B inhibitors, rifampicin, cyclosporine and probenecid. Predictions were done with

in vitro OATP1B Ki values implemented in inhibitor models and Simcyp CP-I model. Interactions with all three inhibitors, rifampicin, cyclosporine, and probenecid. Colours (black, orange, green) represent OATP1B inhibitors (rifampicin, cyclosporine, probenecid). Circles and triangles represent CP-I $C_{max}R$ and AUCR, respectively. Symbols are presented as mean ± standard deviation. Solid, dashed and dotted lines on the graphs represent the line of unity, Guest acceptance criterion and 2-fold error, respectively.

The application of *in vivo* Ki values estimated using Simcyp CP-I model to predict in OATP1B-mediated DDIs with rosuvastatin and pitavastatin is shown in Figure S5. The use of *in vivo* Ki values resulted in comparable prediction of rifampicin interactions to the harmonized model (86%), whereas performance of cyclosporine DDI was poorer (63% within the Guest criterion).

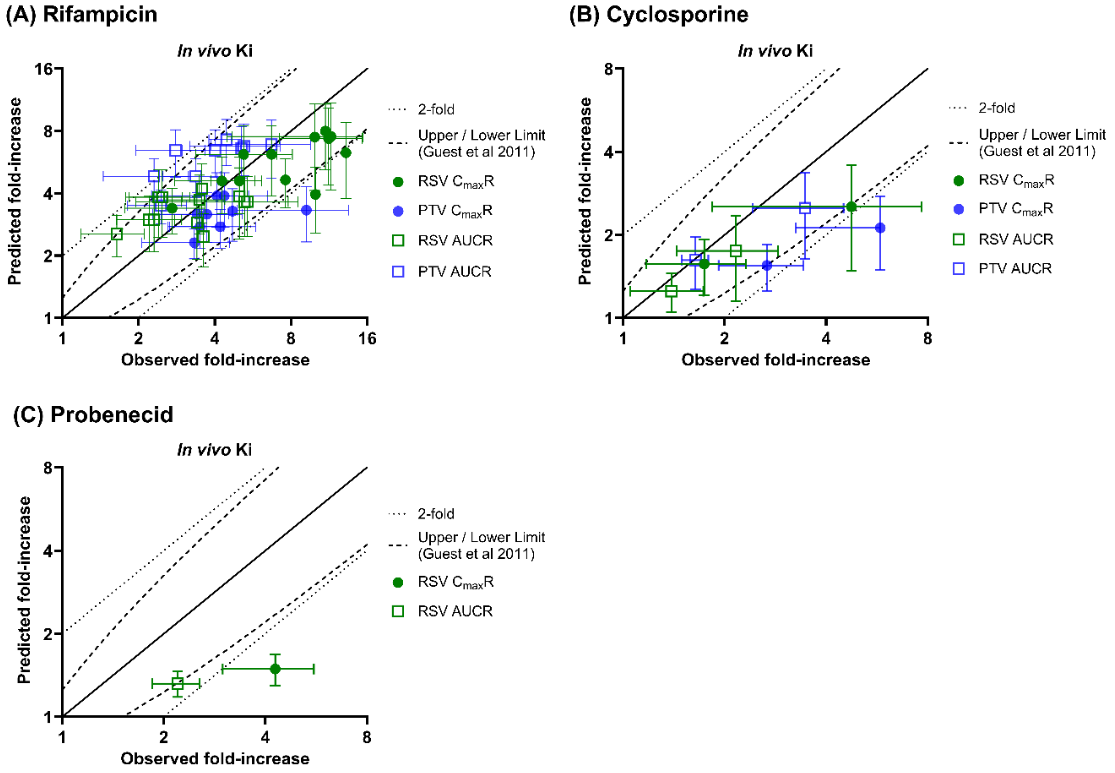

Figure S5. Observed and predicted changes in rosuvastatin/pitavastatin $C_{max}R$ and AUCR after the administration of (A) rifampicin (150-600 mg), (B) cyclosporine (20-75 mg), or (C) probenecid (1000 mg) using *in vivo* Ki from the Simcyp default CP-I model.

Blue and green represent pitavastatin and rosuvastatin. Circles and squares represent $C_{max}R$ and AUCR, respectively. Symbols are presented as mean ± standard deviation. Solid and dashed lines represent the line of unity, 2-fold error and Guest acceptance criterion,[13] respectively.

Supplementary Tables

Table S1. Summary of the reported CP-I plasma baseline with identified ethnicity, *SLCO1B1* c.521T>C genotype and sex.

Ethnicity	Sex	Genotype	N	Age range (year)	$C_{baseline}$ (nM)	Reference
White	M	TT	4	18-45	0.7 ± 0.1	[14]
		TC	3		1.1 ± 0.1	
		CC	1		1.7	
	F	TT	4		0.5 ± 0.1	
		TC	3		0.7 ± 0.1	
		CC	1		1.4	
	M, F	TT (*1/*1)	116	24.1 (mean)	0.8 ± 0.2	[15]
	M, F	TT (*1/*37)	47		0.9 ± 0.2	
	M	TT (*37/*37)	3		0.8 ± 0.2	
	M (n=2), F (n=4)	TC (*1/*5)	6		0.9 ± 0.3	
	M, F	TC (*1/*15)	69		0.9 ± 0.3	
	M (n=7), F (n=10)	TC (*37/*15)	17		0.9 ± 0.3	
	M (n=1), F (n=1)	CC (*5/*15)	2		1.7 ± 0.5	
	M (n=4), F (n=7)	CC (*15/*15)	11		1.4 ± 0.4	
Asian-Indian	M	TT	12	18-45	0.9 ± 0.2	[16]
	M	TT	13	18-45	1.1 ± 0.3	[17]
		TC	1		1.9	
Japanese	M	TT	6	20-45	0.6 ± 0.1	[18]
		TC	5		0.7 ± 0.1	
		CC	2		1.3 ± 0.1	
	F	TT	4		0.5 ± 0.1	
		TC	2		0.6 ± 0.1	
	M	TT	6	26-36	0.8 [a]	[19]
		TC	2		0.9 [a]	
	M (n=32), F (n=71)	TT (*1b/*1b)	103	40-74	0.7 ± 0.2	[20]
	M (n=37), F (n=85)	TT (*1a/*1b)	122	39-74	0.7 ± 0.2	
	M (n=12), F (n=28)	TT (*1a/*1a)	40	39-72	0.7 ± 0.3	
	M (n=17), F (n=57)	TC (*1b/*15)	74	40-73	0.8 ± 0.2	
	M (n=10), F (n=31)	TC (*1a/*15)	41	39-74	0.8 ± 0.2	
	M (n=4), F (n=7)	CC (*15/*15)	11	41-68	1.1 ± 0.5	
	M	TT (n=6), TC (n=2)	8	26-36	0.7 ± 0.2	[21]

Baseline data are presented as mean ± standard deviation. CP-I, $C_{baseline}$, CP-I baseline plasma concentration; Genotype, *SLCO1B1* c.521 T>C genotype.

[a] The values were calculated by using digitized CP-I mean profile.

Table S2. Summary of the clinically reported values of CP-I CL_R.

Ethnicity	Sex	N	CL_R (L/h)	Notes	Reference
Asian-Indian	Male	12	1.9	Observed value with RSV administration	[16]
Asian-Indian	Male	14	1.1	Observed value at predose	[22]
White	Male (n=3), Female (n=3)	6	2.4	Observed value with microdose cocktail administration (MDZ, DABE, PTV, RSV, ATV), healthy volunteer	[23]
White (n=12), Black or African American (n=5)	Male (n=11), Female (n=6)	17	3.0	Observed value with PTV administration	[11]
White	Female	6	2.4	Observed value with Janssen NCE compound administration	[24]
Japanese	Male	10	2.8	Observed value in control group	[25]
**-**		**65**	**2.3**	**The weighted mean of observed CL_R**	**-**

RSV, rosuvastatin; MDZ, midazolam; DABE, dabigatran etexilate; PTV, pitavastatin; ATV, atorvastatin; NCE, new chemical entity

Table S3. Summary of the reported CP-I $C_{max}R$ and AUCR after the administration of rifampicin, cyclosporine, and probenecid.

Inhibitors	Dose (mg), regimen (p.o.)	Ethnicity	Sex	Genotype	N	Age range (year)	CP-I $C_{max}R$	CP-I AUCR	Reference
Rifampicin	600, SD	White	M	N.R.	3	50-71	4.8 ± 0.2	3.3 ± 0.4	[26]
	600, SD		M	N.R.	3	26-47	6.7 ± 1.2	3.7 ± 0.6	[23]
	150, SD	Japanese	M	TT	6	26-36	3.0 [a]	1.6 [a]	[27]
	300, SD						3.6 [a]	2.4 [a]	
	600, SD						6.6 [a]	3.8 [a]	
	150, SD			TC	2		2.0 [a]	1.5 [a]	
	300, SD						3.4 [a]	2.3 [a]	
	600, SD						4.9 [a]	3.3 [a]	
	300, SD		M	TT (n=6), TC (n=2)	8	26-36	3.5 [a]	3.0 ± 0.6	[21]
	600, SD						5.0 [a]	4.6 ± 0.6	
	600, SD	Asian-Indian	M	TT	12	18-45	6.3	4.0 ± 0.5	[16]
Cyclosporine	100, twice daily	White	M	TT	4	18-45	3.0 ± 0.6	1.7 ± 0.2	[14]
				TC	3		2.3 ± 0.5	1.7 ± 0.3	
				CC	1		2.2	1.7	
			F	TT	4		2.7 ± 0.9	1.8 ± 0.4	
				TC	3		3.1 ± 0.1	1.5 ± 0.3	
				CC	1		2.0	1.4	
	20, SD	Japanese	M	N.R.	10	22-39	1.7 ± 0.2	1.2	[28]
	75, SD						3.5 ± 0.3	2.0	
Probenecid	500, QID	White	F	N.R.	6	48-58	1.4	1.4	[24]
	1000, SD	Asian-Indian	M	N.R.	14	18-45	1.5 ± 0.4	1.4 ± 0.2	[22]

$C_{max}R$ and AUCR are presented as mean ± standard deviation. AUCR, area under the plasma-concentration time profile ratio; $C_{max}R$, maximum plasma concentration ratio; N.R., not reported; SD, single dose; QID, quarter in die/ 4 times per day. [a] The values were calculated by using digitized CP-I mean profile.

Table S4. Comparison of CP-I PBPK model parameters developed in this study with other models.

Input/fixed Parameters (unit)		Harmonized model	Kimoto et al [7] / Lin et al [29]	Simcyp default model [i]	Takita et al [3]	Yoshikado et al [4]	Mochizuki et al [22]
$f_{u\ plasma}$		0.069 [3, a]	0.007 [4, a]	0.007 [4, a]	0.069 [3, a]	0.007 [4, a]	0.007 [4, a]
CL_R (L/h)		2.3 [b]	1.9 [16]	1.0	2.7 (estimated)	1.9 [16]	1.9 [16]
Biosynthesis rate (mg/day/kg)	White, Asian-Indian	M: 0.0036 [3]<br>F: 0.0032 [3]	Kimoto et al: 0.0065 [4]<br>Lin et al: 0.0039 [h]	0.0065 [7]	M: 0.0036 (estimated)<br>F: 0.0032 (estimated)	0.0033 - 0.0069 [l]<br>(estimated)	0.0030<br>(estimated)
	Japanese	M: 0.0024 [c,d]<br>F: 0.0021 [c,d]					
CL_{pd} (µL/min/10^6 cells)		0.76[3, a]	1.5 [7, a]		0.76 [3, a]	Not applicable [m]	Not applicable [m]
OATP1B1 CL_{int} (µL/min/million cells)		106 [e] (RAF = 1)	26.3 (RAF = 57) [7]	1160 (RAF = 1) [7,30]	Not applicable [j]		
OATP1B3 CL_{int} (µL/min/million cells)		31 [e] (RAF = 1)	Not reported	339 (RAF = 1) [7,30]			
MRP2 CL_{int} (µL/min/million cells)		0.612 [f] (RAF = 1)	6.5 (RAF = 10) [7]	6.5 (RAF = 10) [7]			
$fT_{OATP1B1}$ (%)		66 [g]	95 [g]	71 [g]			
$fT_{OATP1B3}$ (%)		24 [g]	Not applicable	26 [g]			
**Scaled/ Simulated Parameters (unit)**		**Harmonized model**	**Kimoto et al [7] / Lin et al [29]**	**Simcyp default model [i]**	**Takita et al [3]**	**Yoshikado et al [4]**	**Mochizuki et al [22]**
CL_{active} (L/h) [o]		1500 [g]	16000 [g]	17000 [g]	1400 [3]	Not applicable [n]	
CL_{total} (L/h)		24 [g]	37 [g]	36 [g]	26 [k]	20 – 42 [k]	20 [k]
CL_h (L/h)		21 [g]	35 [g]	35 [g]	23	18 - 40	18
fe (%)		10	4.7	2.7	11	4.4 - 9.1	9.2

B/P, blood to plasma ratio; CL_{active}, active uptake clearance; CL_h, hepatic clearance; CL_{int}, intrinsic transport clearance; $CL_{passive}$, passive transport clearance; CL_{pd}, passive transport clearance; CL_R, renal clearance; CL_{total}, total clearance: fe, fraction excreted in urine; $fT_{OATP1B1}$, fraction transported via OATP1B1 in liver; $fT_{OATP1B3}$, fraction transported via OATP1B3 in liver; $fT_{passive}$, fraction transported via passive diffusion in liver; $f_{u\ plasma}$, fraction unbound in plasma; Mol, molecular; MRP2, multidrug resistance-associated protein 2; OATP1B1/3, organic anion-transporting polypeptide (OATP) 1B1/3; Passive, passive Transport; PBPK, physiologically based pharmacokinetics.

[a] The parameters were fixed based on *in vitro* data. [b] Clinically observed weighted mean values of CP-I CL_R (Table S2). [c] Adjusted biosynthesis rate to recover the clinical data. [d] The sex ratio of k_{syn} was incorporated.[3] [e] CL_{active}[3] was converted to OATP1B CL_{int}. The reported contribution of OATP1B1 and OATP1B3 to hepatic uptake of CP-I[30] were considered. [f] CL_{bile}[3] was converted to MRP2 CL_{int}. [g] Predicted mean values using Simcyp Sim-NEurCaucasian male population (age: 20-50) for a population size of 100 (10 trials * 10 subjects). [h] Adjusted biosynthesis rate to recover the clinical data. Synthesis rate is a function of body weight. [i] Simcyp default model “EB-Coproporphyrin I” in Simcyp library (version 23). [j] The model adopted top-down approach, and CL_{active} and CL_{bile} were estimated. [k] Calculated as $k_{syn}/C_{baseline}$
[l] The range under the conditions of β = 0.2, 0.5, and 0.8. [m] The model defined $PS_{dif,inf}$, $PS_{dif,eff}$, $PS_{act,inf}$, $CL_{int,bile}$ and $CL_{int,met}$ as input parameters, but individual values are not reported. The contribution of OATP1B1 and OATP1B3 were not considered. [n] Model parameterization does not allow derivation of CL_{active}. [o] Based on unbound concentration.

Table S5. Input parameters of rifampicin PBPK model

Parameters (unit)	Value
Mol Weight (g/mol)	823
log P	4.01
Compound Type	Ampholyte
pKa1	1.7
pKa2	7.9
B/P	0.9
Fraction unbound in plasma	0.116
Absorption Model	ADAM
f_a	0.948
$f_{u,gut}$	1
Caco2 Papp (10^{-6} cm/s)	5
Distribution model	Full PBPK Model
Prediction method	Method 2 (Rodgers and Rowland method)
V_{ss} (L/kg)	0.5
K_p Scalar	0.12
Additional HLM CL_{int} (μL/min/mg protein)	0.9
Biliary CL_{int} (Hep) (μL/min/10^6)	0.3
Active Hepatic Scalar (Net)	1
CL_R (L/h)	1.26
CL_{pd} (μL/min/10^6 cells)	0.01
OATP1B1 $K_{i,unbound}$ (μM)	0.162
OATP1B3 $K_{i,unbound}$ (μM)	0.088

f_a, fraction absorbed; $f_{u,gut}$, fraction unbound in the enterocytes; HLM, human liver microsome; $K_{i,unbound}$, unbound inhibition constant; K_p, tissue:plasma partition coefficient; K_p Scalar, scalar applied to all predicted tissue K_p values; Mol, molecular; MRP2, multidrug resistance-associated protein 2; OATP1B1/3, organic anion-transporting polypeptide (OATP) 1B1/3; V_{ss}, volume of distribution at steady-state
Input parameters were obtained from Simcyp default file (SV-Rifampicin) in Simcyp library.

Table S6. Input parameters of cyclosporine PBPK model

Parameters (unit)	Value
Mol Weight (g/mol)	1202
log P	2.96
Compound Type	Neutral
B/P	1.62
Fraction unbound in plasma	0.037
Absorption Model	First Order
f_a	1
k_a (1/h)	1.659
$f_{u,gut}$	1
$Caco_2$ Papp (10^{-6} cm/s)	17
Distribution model	Full PBPK Model
Prediction method	Method 2 (Rodgers and Rowland method)
V_{ss} (L/kg)	1.77
K_p Scalar	1.2
Active Hepatic Scalar (Net)	1.53
Biliary CL_{int} (Hep) (uL/min/10^6)	0.45
CL_R (L/h)	0.029
OATP1B1 $K_{i,unbound}$ (μM)	0.019
OATP1B3 $K_{i,unbound}$ (μM)	0.032

Input parameters were obtained from Simcyp default file (SV-Cyclosporine_Neoral) in Simcyp library.

Table S7. Input parameters of probenecid PBPK model

Parameters (unit)	Value
Mol Weight (g/mol)	285.36
log P	3.21
Compound Type	Monoprotic Acid
B/P	0.55
Fraction unbound in plasma	0.100
Absorption Model	ADAM
$P_{eff,man}$ (10^{-4} cm/s)	1.73
f_a	0.899
k_a (1/h)	0.755
PSA (Å)	85.24
$f_{u,gut}$	1
Distribution model	Full PBPK Model
Prediction method	Method 2 (Rodgers and Rowland method)
V_{ss} (L/kg)	0.111
K_p Scalar	1
HLM V_{max} (pmol/min/mg protein)	261.8
HLM K_m (µM)	76.8
Active Hepatic Scalar (Net)	1
CL_R (L/h)	0.09
OAT1/3 $K_{i,unbound}$ (µM)	3.4
OATP1B1 $K_{i,unbound}$ (µM)	167 [a]
OATP1B3 $K_{i,unbound}$ (µM)	76 [a]

OAT1/3, organic anion transporter 1/3; OATP1B1/3, $P_{eff,man}$, human jejunum effective permeability. Input parameters were obtained from our previous study.[31] [a]IC_{50} values for OATP1B1 and OATP1B3, reported from *in vitro* studies using CP-I as a probe[22] were incorporated.

Table S8. Input parameters of rosuvastatin PBPK model

Parameters (unit)	Value
Mol Weight (g/mol)	481.5
log P	2.4
Compound Type	Monoprotic Acid
B/P	0.63
Fraction unbound in plasma	0.11
Absorption Model	ADAM
$P_{eff,man}$ (10^{-4} cm/s)	0.16
$f_{u,gut}$	1
Distribution model	Full PBPK Model
Prediction method	Method 2 (Rodgers and Rowland method)
V_{ss} (L/kg)	0.12
K_p Scalar	1
HLM CL_{int} (μL/min/mg protein)	3.2
Active Hepatic Scalar (Net)	1
CL_R (L/h)	13.6
**Permeability Limited Liver Model**	
CL_{pd} (μL/min/10^6 cells)	1.4
NTCP CL_{int} (μL/min/10^6 cells)	13.2
OATP1B1 CL_{int} (μL/min/10^6 cells)	130
OATP1B3 CL_{int} (μL/min/10^6 cells)	26.5
OATP2B1 CL_{int} (μL/min/10^6 cells)	46.4
MRP4 CL_{int} (μL/min/10^6 cells)	6.46
BCRP CL_{int} (μL/min/10^6 cells)	6.46

BCRP, breast cancer resistance protein; NTCP, Na+-taurocholate co-transporting polypeptide; MRP4, multidrug resistance-associated protein 4; OATP2B1, organic anion-transporting polypeptide (OATP) 2B1. Input parameters were obtained from Simcyp default file (SV-Rosuvastatin) in Simcyp library.

Table S9. Input parameters of pitavastatin PBPK model

Parameters (unit)	Value
Mol Weight (g/mol)	421.47
log P	2.75
Compound Type	Monoprotic Acid
B/P	0.58
Fraction unbound in plasma	0.004
Absorption Model	ADAM
$P_{eff,man}$ (10^{-4} cm/s)	1.02
$f_{u,gut}$	1
Distribution model	Full PBPK Model
Prediction method	Method 2 (Rodgers and Rowland method)
V_{ss} (L/kg)	0.094
K_p Scalar	1
Active Hepatic Scalar (Net)	1
CL_{bile} (L/h)	5.4
CL_R (L/h)	0.6
**Permeability Limited Liver Model**	
CL_{pd} (µL/min/10^6 cells)	6.72
NTCP CL_{int} (µL/min/10^6 cells)	79.29
OATP1B1 CL_{int} (µL/min/10^6 cells)	1224.7
OATP1B3 CL_{int} (µL/min/10^6 cells)	168.21
OATP2B1 CL_{int} (µL/min/10^6 cells)	206

Input parameters were obtained from compound repository in Simcyp members area (RES-Pitavastatin).

Table S10. Summary of *in vitro* and estimated/reported *in vivo* Ki values based on CP-I interaction profiles for OATP1B inhibitors.

Inhibitor	*In vitro* OATP1B $K_{i,u}$ (μM)		Note
Rifampicin	0.162		Ki values in Simcyp default file (SV-Rifampicin)
Cyclosporine	0.019		Ki values in Simcyp default file (SV-Cyclosporine_Neoral)
Probenecid	167		*In vitro* IC50 reported by Zhang et al.[22] using CP-I as a probe
**Inhibitor**	**Inhibitor dose (mg)** [a]	***In vivo* OATP1B $K_{i,u}$ (μM)**	**Reference**
Rifampicin	600	0.053 [b]	Estimated based on the harmonized CP-I model
	600	0.11 [b]	Estimated based on Simcyp default CP-I model
	600	0.13	[10]
	600	0.020	[9]
	300 - 600	0.082 - 0.11	[4]
	600	0.10	[3]
	150 - 600	0.14 [b]	[7]
	600	0.038	[32]
Cyclosporine	100	0.0021 [b]	Estimated based on harmonized CP-I model
	100	0.0025 [b]	Estimated based on Simcyp default CP-I model
	100	0.014 [b]	[7]
	20 - 75	0.00054	[28]
Probenecid	1000	30 [b]	Estimated based on harmonized CP-I model
	1000	33 [b]	Estimated based on Simcyp default CP-I model
	1000	19 [b]	[7]

[a] The dose of the inhibitor used for the estimation of *in vivo* Ki. [b] $K_{i,u,OATP1B1}$; OATP1B1 and OATP1B3 transporters were defined separately.

Table S11. Summary of the reported rosuvastatin and pitavastatin interactions after the administration of rifampicin, cyclosporine, and probenecid.

Inhibitors	Inhibitor dose (mg)	Substrates	Substrate dose (mg)	Ethnicity	Sex	Genotype	N	Age	$C_{max}R$	AUCR	Reference
Rifampicin	150	Rosuvastatin							2.7	1.6	
	300		0.5	Japanese	M	TT (n=6), TC (n=2)	8	26-36	4.3	2.3	[27]
	600								5.2	2.5	
	300		0.5	Japanese	M	TT (n=6), TC (n=2)	8	26-36	5.0	2.2	[21]
	600								6.7	2.4	
	600		5	Asian-Indian	M	TT	11	18-45	13	5.0	[16]
	600		0.05	White	M (n=3), F (n=3)	N.R.	6	50-71	11	3.6	[23]
	600		20	White	M (n=4), F (n=3)	N.R.	7	18-65	7.6	3.6	[33]
	600		20	Asian	M (n=3), F (n=5)	N.R.	8	18-65	10	3.4	[33]
	600		10	White, Asian [a,c]	M	N.R.	12	18-55	11	3.5	[34]
	600		5	N.R.[b,c]	M, F [d]	N.R.	8	19-55	9.9	5.2	[35]
	600		0.025	N.R.[c]	M, F [d]	N.R.	7	19-55	12	5.4	[36]
	150	Pitavastatin							3.3	2.5	
	300		0.2	Japanese	M	TT (n=6), TC (n=2)	8	26-36	4.2	3.4	[27]
	600								3.7	4.0	
	300		0.2	Japanese	M	TT (n=6), TC (n=2)	8	26-36	3.5	2.3	[21]
	600								3.4	2.8	
	600		0.2	Japanese	M	N.R.	8	20-45	4.7	5.1	[37]
	600		0.01	White	M (n=3), F (n=3)	N.R.	6	50-71	3.7	3.7	[23]
	600		4	Asian	M	N.R.	12	24.1 (mean)	9.2	6.7	[38]
	600		1	N.R. [b,c]	M, F [d]	N.R.	8	19-55	4.4	5.2	[35]
	600		0.01	N.R. [c]	M, F [d]	N.R.	7	19-55	4.1	4.5	[36]
Cyclosporine	20	Rosuvastatin	1	Japanese	M	N.R.	10	22-39	1.7	1.4	[28]
	75								4.8	2.2	
	20	Pitavastatin	0.2						2.7	1.6	
	75								5.8	3.5	
Probenecid	1000	Rosuvastatin	10	White, Asian [a,c]	M	N.R.	16	18-55	4.3	2.2	[34]

AUCR, area under the plasma-concentration time profile ratio; $C_{max}R$, maximum plasma concentration ratio. [a] The clinical study consisted of three parts (Part 1, 2 and 3), evaluating different transporter inhibitors. A total of 45 subjects participated, comprising 44 White and 1 Asian. Each part had a different number of participants: Part 1 included 12 subjects, Part 2 had 17 subjects, and Part 3 involved 16 subjects. The clinical trial where rifampicin or probenecid were administered corresponds to Part 1 or 3, respectively. Detailed ethnicity data were not disclosed. [b] The clinical study was conducted in the United States, but the ethnicity of the subjects was not reported. [c] Sim-NEurCaucasian population was used for simulations. [d] Simulations assumed 50% male and 50% female contribution.

Table S12. The characteristics of the virtual OATP1B inhibitors with potency ranging from weak to strong OATP1B and either short or long $t_{1/2}$.

Virtual OATP1B inhibitor	OATP1B $K_{i,u}$ (µM)	Inhibitor $C_{max,u}$ / OATP1B $K_{i,u}$	Inhibitor classification
(a) Strong inhibitor with short $t_{1/2}$	0.1	68	AUC or C_{max} changes ≥ 5-fold
(b) Moderate inhibitor with short $t_{1/2}$	1	7	AUC or C_{max} changes ≥ 2- to < 5-fold
(c) Weak inhibitor with short $t_{1/2}$	10	0.7	AUC or C_{max} changes ≥ 1.25- to < 2-fold
(d) Strong inhibitor with long $t_{1/2}$	0.1	31	AUC or C_{max} changes ≥ 5-fold

$C_{max,u}$, unbound maximum plasma concentration; $K_{i,u}$, unbound inhibition constant; $t_{1/2}$, half life

Table S13. Summary of observed versus predicted CP-I plasma baseline using the harmonized CP-I PBPK model

Ethnicity	Sex	Genotype	N	$C_{baseline}$ (nM) Pred.	$C_{baseline}$ (nM) Obs.	$R_{pred/obs}$	Reference for observed data
White	M	TT	4	0.9 ± 0.4	0.7 ± 0.1	1.4	14
		TC	3	1.1 ± 0.4	1.1 ± 0.1	1.0	
		CC	1	1.3 ± 0.5	1.7	0.8	
	F	TT	4	0.7 ± 0.3	0.5 ± 0.1	1.3	
		TC	3	0.8 ± 0.3	0.7 ± 0.1	1.2	
		CC	1	1.0 ± 0.3	1.4	0.7	
	M, F [a]	TT (*1/*1)	116	0.9 ± 0.4	0.8 ± 0.2	1.0	15
	M, F [a]	TT (*1/*37)	47	0.9 ± 0.4	0.9 ± 0.2	1.0	
	M	TT (*37/*37)	3	1.0 ± 0.4	0.8 ± 0.2	1.1	
	M (n=2), F (n=4)	TC (*1/*5)	6	0.9 ± 0.4	0.9 ± 0.3	1.0	
	M, F [a]	TC (*1/*15)	69	1.0 ± 0.4	0.9 ± 0.3	1.1	
	M (N=7), F (N=10)	TC (*37/*15)	17	1.0 ± 0.4	0.9 ± 0.3	1.0	
	M (N=1), F (N=1)	CC (*5/*15)	2	1.2 ± 0.4	1.7 ± 0.5	0.7	
	M (n=4), F (n=7)	CC (*15/*15)	11	1.2 ± 0.4	1.4 ± 0.4	0.9	
Asian-Indians	M	TT	12	1.1 ± 0.4	0.9 ± 0.2	1.3	16
	M	TT	13	1.1 ± 0.4	1.1 ± 0.3	1.0	17
		TC	1	1.2 ± 0.5	1.9	0.7	
Japanese	M	TT	6	0.7 ± 0.3	0.6 ± 0.1	1.1	18
		TC	5	0.8 ± 0.3	0.7 ± 0.1	1.2	
		CC	2	1.0 ± 0.4	1.3 ± 0.1	0.8	
	F	TT	4	0.5 ± 0.2	0.5 ± 0.1	1.1	
		TC	2	0.6 ± 0.3	0.6 ± 0.1	1.0	
	M	TT	6	0.7 ± 0.3	0.8	0.9	27
		TC	2	0.8 ± 0.3	0.9	1.0	
	M (n=32), F (n=71)	TT (*1b/*1b)	103	0.8 ± 0.4	0.7 ± 0.2	1.1	20
	M (n=37), F (n=85)	TT (*1a/*1b)	122	0.8 ± 0.4	0.7 ± 0.2	1.0	
	M (n=12), F (n=28)	TT (*1a/*1a)	40	0.8 ± 0.3	0.7 ± 0.3	1.0	
	M (n=17), F (n=57)	TC (*1b/*15)	74	0.8 ± 0.3	0.8 ± 0.2	1.0	
	M (n=10), F (n=31)	TC (*1a/*15)	41	0.8 ± 0.3	0.8 ± 0.2	1.0	
	M (n=4), F (n=7)	CC (*15/*15)	11	1.0 ± 0.4	1.1 ± 0.5	0.9	
	M	TT (n=6), TC (n=2)	8	0.7 ± 0.3	0.7 ± 0.2	1.0	21

Ethnicity	Sex	Genotype	N	$C_{baseline}$ (nM) Pred.	$C_{baseline}$ (nM) Obs.	Female/male ratio [b] Pred.	Female/male ratio [b] Obs.	Reference

White	M	TT	4	0.9 ± 0.4	0.7 ± 0.1	-	-	14
	F	TT	4	0.7 ± 0.3	0.5 ± 0.1	0.8	0.8	
Japanese	M	TT	6	0.7 ± 0.3	0.6 ± 0.1	-	-	18
	F	TT	4	0.5 ± 0.2	0.5 ± 0.1	0.8	0.8	

Ethnicity	Sex	Genotype	N	$C_{baseline}$ (nM)		Ratio $_{CC/TT}$ [c]		Reference
				Pred.	Obs.	Pred.	Obs.	
White	M, F [a]	TT (*1/*1)	116	0.9 ± 0.4	0.8 ± 0.2	-	-	15
	M (n=4), F (n=7)	CC (*15/*15)	11	1.2 ± 0.4	1.4 ± 0.4	1.4	1.6	
Japanese	M (n=32), F (n=71)	TT (*1b/*1b)	103	0.8 ± 0.4	0.7 ± 0.2	-	-	20
	M (n=4), F (n=7)	CC (*15/*15)	11	1.0 ± 0.4	1.1 ± 0.5	1.4	1.6	

Data are presented as mean ± standard deviation; $C_{baseline}$, CP-I baseline plasma concentration; GMFE, geometric mean fold error; $R_{obs/pred}$, Ratio of prediction to observation. [a] The number of males and females was not disclosed in the study and simulations assumed 50% male and 50% female. [b] The baseline ratio of female to male. [c] The baseline ratio of CC to TT subjects.

Table S14. Summary of observed versus predicted CP-I $C_{max}R$ and AUCR after administration of rifampicin, cyclosporine, and probenecid using the harmonized CP-I PBPK model

Inhibitors	Dose (mg)	Ethnicity	Sex	Genotype	Metric	Pred.	Obs.	$R_{pred/obs}$	Reference for observed data
Rifampicin	600	White	M	N.R.	$C_{max}R$	3.6 ± 0.7	4.8 ± 0.2	0.8	26
	600	White	M	N.R.		3.4 ± 0.7	6.7 ± 1.2	0.5	23
	150	Japanese	M	TT		2.1 ± 0.3	3.0	0.7	27
	300					2.7 ± 0.5	3.6	0.8	
	600					3.6 ± 0.8	6.6	0.5	
	150			TC		2.2 ± 0.3	2.0	1.1	
	300					2.9 ± 0.5	3.4	0.8	
	600					3.7 ± 0.9	4.9	0.8	
	300	Japanese	M	TT (n=6), TC (n=2)		2.7 ± 0.5	3.5	0.8	21
	600		M			3.6 ± 0.8	5.0	0.7	
	600	Asian-Indian	M	TT		3.5 ± 0.8	6.3	0.6	16
	600	White	M	N.R.	AUCR	2.1 ± 0.6	3.3 ± 0.4	0.6	26
	600	White	M	N.R.		1.9 ± 0.5	3.7 ± 0.6	0.5	23
	150	Japanese	M	TT		1.4 ± 0.3	1.6	0.9	27
	300					1.7 ± 0.4	2.4	0.7	
	600					2.2 ± 0.6	3.8	0.6	
	150			TC		1.5 ± 0.2	1.5	1.0	
	300					1.8 ± 0.4	2.3	0.8	
	600					2.4 ± 0.6	3.3	0.7	
	300	Japanese	M	TT (n=6), TC (n=2)		1.7 ± 0.4	3.0 ± 0.6	0.6	21
	600					2.2 ± 0.6	4.6 ± 0.6	0.5	
	600	Asian-Indian	M	TT		2.2 ± 0.6	4.0 ± 0.5	0.5	16
Cyclosporine	100	White	M	TT	$C_{max}R$	1.4 ± 0.1	3.0 ± 0.6	0.5	14
				TC		1.4 ± 0.2	2.3 ± 0.5	0.6	
				CC		1.4 ± 0.2	2.2	0.6	
			F	TT		1.5 ± 0.2	2.7 ± 0.9	0.6	
				TC		1.5 ± 0.2	3.1 ± 0.1	0.5	
				CC		1.4 ± 0.2	2.0	0.7	
	20	Japanese	M	N.R.		1.1 ± 0.0	1.7 ± 0.2	0.6	28
	75			N.R.		1.3 ± 0.1	3.5 ± 0.3	0.4	
	100	White	M	TT	AUCR	1.1 ± 0.0	1.7 ± 0.2	0.7	14
				TC		1.1 ± 0.1	1.7 ± 0.3	0.7	
				CC		1.1 ± 0.1	1.7	0.7	
			F	TT		1.1 ± 0.1	1.8 ± 0.4	0.6	
				TC		1.2 ± 0.1	1.5 ± 0.3	0.8	

				CC		1.1 ± 0.1	1.4	0.8	
	20	Japanese	M	N.R.		1.0 ± 0.0	1.2	0.8	28
	75	Japanese	M	N.R.		1.1 ± 0.1	2.0	0.6	28
Probenecid	500	White	F	N.R.	$C_{max}R$	1.2 ± 0.0	1.4	0.9	24
Probenecid	1000	Asian-Indian	M	N.R.	$C_{max}R$	1.2 ± 0.0	1.5 ± 0.4	0.7	22
Probenecid	500	White	F	N.R.	AUCR	1.2 ± 0.1	1.4	0.8	24
Probenecid	1000	Asian-Indian	M	N.R.	AUCR	1.1 ± 0.0	1.4 ± 0.2	0.8	22

Inhibitor	Dose (mg)	Ethnicity	Sex	Genotype	Metric	Mean range [a]		-	Reference
						Pred.	Obs.		
Rifampicin	600	White, Japanese, Asian-Indian	M, F	TT, TC	$C_{max}R$	3.4-3.7	4.8-6.7	-	16,21,23,26,27
					AUCR	1.9-2.4	3.3-4.6		
Cyclosporine	100	White	M,F	TT, TC, CC	$C_{max}R$	1.4-1.5	2.0-3.1		14
					AUCR	1.1-1.2	1.4-1.8		
Probenecid	1000	Asian-Indian	M	N.R.	$C_{max}R$	1.2	1.5		22
					AUCR	1.1	1.4		

Data are presented as mean ± standard deviation. AUCR, area under the plasma-concentration time profile ratio; $C_{max}R$, maximum plasma concentration ratio; N.R., not reported; Obs, observed; Pred, predicted; $R_{pred/obs}$: Ratio of prediction to observation. [a] The range of the mean values for observation and prediction.

REFERENCES

1. Birmingham, B. K. *et al.* Rosuvastatin pharmacokinetics and pharmacogenetics in Caucasian and Asian subjects residing in the United States. *Eur J Clin Pharmacol* **71**, 329–340 (2015).
2. Tomita, Y., Maeda, K. & Sugiyama, Y. Ethnic variability in the plasma exposures of OATP1B1 substrates such as HMG-CoA reductase inhibitors: a kinetic consideration of its mechanism. *Clin Pharmacol Ther* **94**, 37–51 (2013).
3. Takita, H. *et al.* PBPK Model of Coproporphyrin I: Evaluation of the Impact of SLCO1B1 Genotype, Ethnicity, and Sex on its Inter-Individual Variability. *CPT: Pharmacometrics & Systems Pharmacology* **10**, 137–147 (2021).
4. Yoshikado, T. *et al.* PBPK Modeling of Coproporphyrin I as an Endogenous Biomarker for Drug Interactions Involving Inhibition of Hepatic OATP1B1 and OATP1B3. *CPT: Pharmacometrics & Systems Pharmacology* **7**, 739–747 (2018).
5. Gu, X. *et al.* Absorption and Disposition of Coproporphyrin I (CPI) in Cynomolgus Monkeys and Mice: Pharmacokinetic Evidence to Support the Use of CPI to Inform the Potential for Organic Anion-Transporting Polypeptide Inhibition. *Drug Metab Dispos* **48**, 724–734 (2020).
6. Aziz, M. A. & Watson, C. J. An analysis of the porphyrins of normal and cirrhotic human liver and normal bile. *Clinica Chimica Acta* **26**, 525–531 (1969).
7. Kimoto, E. *et al.* Biomarker-Informed Model-Based Risk Assessment of Organic Anion Transporting Polypeptide 1B Mediated Drug-Drug Interactions. *Clinical Pharmacology & Therapeutics* **111**, 404–415 (2022).
8. Koskelo, P. & Kekki, M. Multicompartment analysis of 14C-labelled coproporphyrin and uroporphyrin kinetics in human beings. *Ann Clin Res* **8 Suppl 17**, 198–202 (1976).
9. Yoshida, K., Guo, C. & Sane, R. Quantitative Prediction of OATP-Mediated Drug-Drug Interactions With Model-Based Analysis of Endogenous Biomarker Kinetics. *CPT: Pharmacometrics & Systems Pharmacology* **7**, 517–524 (2018).
10. Barnett, S. *et al.* Gaining Mechanistic Insight Into Coproporphyrin I as Endogenous Biomarker for OATP1B-Mediated Drug–Drug Interactions Using Population Pharmacokinetic Modeling and Simulation. *Clinical Pharmacology & Therapeutics* **104**, 564–574 (2018).
11. Feng, S. *et al.* Further Evaluation of Coproporphyrins as Clinical Endogenous Markers for OATP1B. *The Journal of Clinical Pharmacology* **61**, 1027–1034 (2021).

12. Rodrigues, A. d. Reimagining the Framework Supporting the Static Analysis of Transporter Drug Interaction Risk; Integrated Use of Biomarkers to Generate Pan-Transporter Inhibition Signatures. *Clinical Pharmacology & Therapeutics* **113**, 986–1002 (2023).

13. Guest, E. J., Aarons, L., Houston, J. B., Rostami-Hodjegan, A. & Galetin, A. Critique of the Two-Fold Measure of Prediction Success for Ratios: Application for the Assessment of Drug-Drug Interactions. *Drug Metab Dispos* **39**, 170–173 (2011).

14. Yee, S. W. *et al.* Organic Anion Transporter Polypeptide 1B1 Polymorphism Modulates the Extent of Drug-Drug Interaction and Associated Biomarker Levels in Healthy Volunteers. *Clin Transl Sci* **12**, 388–399 (2019).

15. Neuvonen, M., Tornio, A., Hirvensalo, P., Backman, J. T. & Niemi, M. Performance of Plasma Coproporphyrin I and III as OATP1B1 Biomarkers in Humans. *Clinical Pharmacology & Therapeutics* **110**, 1622–1632 (2021).

16. Lai, Y. *et al.* Coproporphyrins in Plasma and Urine Can Be Appropriate Clinical Biomarkers to Recapitulate Drug-Drug Interactions Mediated by Organic Anion Transporting Polypeptide Inhibition. *J Pharmacol Exp Ther* **358**, 397–404 (2016).

17. Shen, H. *et al.* Coproporphyrins I and III as Functional Markers of OATP1B Activity: In Vitro and In Vivo Evaluation in Preclinical Species. *J Pharmacol Exp Ther* **357**, 382–393 (2016).

18. Mori, D. *et al.* Effect of OATP1B1 genotypes on plasma concentrations of endogenous OATP1B1 substrates and drugs, and their association in healthy volunteers. *Drug Metabolism and Pharmacokinetics* **34**, 78–86 (2019).

19. Mori, D. *et al.* Alteration in the Plasma Concentrations of Endogenous Organic Anion–Transporting Polypeptide 1B Biomarkers in Patients with Non–Small Cell Lung Cancer Treated with Paclitaxel. *Drug Metab Dispos* **48**, 387–394 (2020).

20. Suzuki, Y. *et al.* Relationship of hemoglobin level and plasma coproporphyrin-I concentrations as an endogenous probe for phenotyping OATP1B. *Clinical and Translational Science* **14**, 1403–1411 (2021).

21. Takehara, I. *et al.* Comparative Study of the Dose-Dependence of OATP1B Inhibition by Rifampicin Using Probe Drugs and Endogenous Substrates in Healthy Volunteers. *Pharm Res* **35**, 138 (2018).

22. Zhang, Y. *et al.* Detection of Weak Organic Anion–Transporting Polypeptide 1B Inhibition by Probenecid with Plasma-Based Coproporphyrin in Humans. *Drug Metab Dispos* **48**, 841–848 (2020).
23. Tatosian, D. A. *et al.* A Microdose Cocktail to Evaluate Drug Interactions in Patients with Renal Impairment. *Clinical Pharmacology & Therapeutics* **109**, 403–415 (2021).
24. Willemin, M.-E. *et al.* Clinical Investigation on Endogenous Biomarkers to Predict Strong OAT-Mediated Drug–Drug Interactions. *Clin Pharmacokinet* **60**, 1187–1199 (2021).
25. Mochizuki, T. *et al.* Effect of Cyclosporin A and Impact of Dose Staggering on OATP1B1/1B3 Endogenous Substrates and Drug Probes for Assessing Clinical Drug Interactions. *Clinical Pharmacology & Therapeutics* **111**, 1315–1323 (2022).
26. Shen, H. *et al.* Further Studies to Support the Use of Coproporphyrin I and III as Novel Clinical Biomarkers for Evaluating the Potential for Organic Anion Transporting Polypeptide 1B1 and OATP1B3 Inhibition. *Drug Metab Dispos* **46**, 1075–1082 (2018).
27. Mori, D. *et al.* Dose-Dependent Inhibition of OATP1B by Rifampicin in Healthy Volunteers: Comprehensive Evaluation of Candidate Biomarkers and OATP1B Probe Drugs. *Clinical Pharmacology & Therapeutics* **107**, 1004–1013 (2020).
28. Mochizuki, T. *et al.* Physiologically-based pharmacokinetic model-based translation of OATP1B-mediated drug–drug interactions from coproporphyrin I to probe drugs. *Clinical and Translational Science* **15**, 1519–1531 (2022).
29. Lin, J. *et al.* Effect of Hepatic Impairment on OATP1B Activity: Quantitative Pharmacokinetic Analysis of Endogenous Biomarker and Substrate Drugs. *Clinical Pharmacology & Therapeutics* **113**, 1058–1069 (2023).
30. Chan, G. H. *et al.* Evaluation of the selectivity of several OATP1B biomarkers using relative activity factor method. *Drug Metab Dispos* (2023) doi:10.1124/dmd.122.000972.
31. Tan, S. P. F. *et al.* Development of 4-Pyridoxic Acid PBPK Model to Support Biomarker-Informed Evaluation of OAT1/3 Inhibition and Effect of Chronic Kidney Disease. *Clinical Pharmacology & Therapeutics* **114**, 1243–1253 (2023).
32. Takita, H. *et al.* Coproporphyrin I as an Endogenous Biomarker to Detect Reduced OATP1B Activity and Shift in Elimination Route in Chronic Kidney Disease. *Clin Pharmacol Ther* **112**, 615–626 (2022).

33. Wu, H.-F. *et al.* Rosuvastatin pharmacokinetics in Asian and White subjects wild-type for both OATP1B1 and BCRP under control and inhibited conditions. *J Pharm Sci* **106**, 2751–2757 (2017).

34. Wiebe, S. T. *et al.* Validation of a Drug Transporter Probe Cocktail Using the Prototypical Inhibitors Rifampin, Probenecid, Verapamil, and Cimetidine. *Clin Pharmacokinet* **59**, 1627–1639 (2020).

35. Prueksaritanont, T. *et al.* Pitavastatin is a more sensitive and selective organic anion-transporting polypeptide 1B clinical probe than rosuvastatin. *Br J Clin Pharmacol* **78**, 587–598 (2014).

36. Prueksaritanont, T. *et al.* Validation of a microdose probe drug cocktail for clinical drug interaction assessments for drug transporters and CYP3A. *Clinical Pharmacology & Therapeutics* **101**, 519–530 (2017).

37. Kim, S.-J. *et al.* Clarification of the Mechanism of Clopidogrel-Mediated Drug–Drug Interaction in a Clinical Cassette Small-dose Study and Its Prediction Based on In Vitro Information. *Drug Metab Dispos* **44**, 1622–1632 (2016).

38. Chen, Y. *et al.* Effect of a single-dose rifampin on the pharmacokinetics of pitavastatin in healthy volunteers. *Eur J Clin Pharmacol* **69**, 1933–1938 (2013).
